# Risks and Population Burden of Cardiovascular Diseases Associated with Diabetes in China: A Prospective Study of 0.5 Million Adults

**DOI:** 10.1371/journal.pmed.1002026

**Published:** 2016-07-05

**Authors:** Fiona Bragg, Liming Li, Ling Yang, Yu Guo, Yiping Chen, Zheng Bian, Junshi Chen, Rory Collins, Richard Peto, Chunmei Wang, Caixia Dong, Rong Pan, Jinyi Zhou, Xin Xu, Zhengming Chen, China Kadoorie Biobank (CKB) collaborative group

**Affiliations:** 1 Clinical Trial Service Unit and Epidemiological Studies Unit, Nuffield Department of Population Health, University of Oxford, Oxford, United Kingdom; 2 Department of Epidemiology and Biostatistics, School of Public Health, Peking University, Beijing, China; 3 Chinese Academy of Medical Sciences, Beijing, China; 4 China National Center for Food Safety Risk Assessment, Beijing, China; 5 Tongxiang Centre for Disease Control and Prevention, Zhejiang, China; 6 Gansu Centre for Disease Control and Prevention, Gansu, China; 7 Liuzhou Centre for Disease Control and Prevention, Liuzhou, China; 8 Jiangsu Centre for Disease Control and Prevention, Jiangsu, China; 9 Liuyang Centre for Disease Control and Prevention, Hunan, China; University of Cambridge, UNITED KINGDOM

## Abstract

**Background:**

In China, diabetes prevalence is rising rapidly, but little is known about the associated risks and population burden of cardiovascular diseases. We assess associations of diabetes with major cardiovascular diseases and the relevance of diabetes duration and other modifiable risk factors to these associations.

**Methods and Findings:**

A nationwide prospective study recruited 512,891 men and women aged 30–79 y between 25 June 2004 and 15 July 2008 from ten diverse localities across China. During ~7 y of follow-up, 7,353 cardiovascular deaths and 25,451 non-fatal major cardiovascular events were recorded among 488,760 participants without prior cardiovascular disease at baseline. Cox regression yielded adjusted hazard ratios (HRs) comparing disease risks in individuals with diabetes to those without. Overall, 5.4% (*n* = 26,335) of participants had self-reported (2.7%) or screen-detected (2.7%) diabetes. Individuals with self-reported diabetes had an adjusted HR of 2.07 (95% CI 1.90–2.26) for cardiovascular mortality. There were significant excess risks of major coronary event (2.44, 95% CI 2.18–2.73), ischaemic stroke (1.68, 95% CI 1.60–1.77), and intracerebral haemorrhage (1.24, 95% CI 1.07–1.44). Screen-detected diabetes was also associated with significant, though more modest, excess cardiovascular risks, with corresponding HRs of 1.66 (95% CI 1.51–1.83), 1.62 (95% CI 1.40–1.86), 1.48 (95% CI 1.40–1.57), and 1.17 (95% CI 1.01–1.36), respectively. Misclassification of screen-detected diabetes may have caused these risk estimates to be underestimated, whilst lack of data on lipids may have resulted in residual confounding of diabetes-associated cardiovascular disease risks. Among individuals with diabetes, cardiovascular risk increased progressively with duration of diabetes and number of other presenting modifiable cardiovascular risk factors. Assuming a causal association, diabetes now accounts for ~0.5 million (489,676, 95% CI 335,777–681,202) cardiovascular deaths annually in China.

**Conclusions:**

Among Chinese adults, diabetes is associated with significantly increased risks of major cardiovascular diseases. The increasing prevalence and younger age of onset of diabetes foreshadow greater diabetes-attributable disease burden in China.

## Abstract 2

In a large prospective cohort study, Zhengming Chen and colleagues examine the associations between diabetes and major cardiovascular diseases in China where the prevalence of diabetes is rising rapidly.

## Author Summary

**Why Was This Study Done?:**

- In China, approximately one in ten adults now has diabetes, following large and rapid increases in diabetes prevalence over recent decades.
- In Western populations, individuals with diabetes are at higher risk of developing cardiovascular diseases, but relevant evidence is lacking in China, where patterns of cardiovascular disease (e.g., high stroke rates) differ from those in Western populations.
- Even in Western populations, questions remain as to whether diabetes increases the risk of haemorrhagic stroke and how other modifiable risk factors (e.g., smoking, hypertension, overweight or obese) may act together with diabetes to further increase cardiovascular disease risk.

**What Did the Researchers Do and Find?:**

- We recruited 0.5 million Chinese adults to our study in 2004–2008; participants completed a detailed questionnaire interview and underwent physical measurements and blood tests, and we then tracked their health for seven years.
- Individuals with diabetes (previously or newly diagnosed) at the initial baseline assessment had 1.5- to 2.5-fold higher risks of developing ischaemic heart disease and stroke (both ischaemic and haemorrhagic).
- Among individuals with diabetes, cardiovascular disease risks increased progressively with the number of other modifiable cardiovascular disease risk factors present (e.g., smoking, overweight or obese, hypertension, and low physical activity) and with a longer duration of diabetes diagnosis.

**What Do These Findings Mean?:**

- These findings show that Chinese adults with diabetes have a substantially higher risk of cardiovascular diseases than those without diabetes.
- Based on these findings, we estimate that diabetes now accounts for almost 0.5 million cardiovascular disease deaths each year in China; this disease burden will likely increase as diabetes is expected to become more prevalent in China over coming decades, especially among young adults.
- To help to reduce the risk of cardiovascular diseases, individuals with diabetes should not only take necessary medications, but also improve their lifestyles, e.g., through smoking cessation, weight loss, and increased physical activity.

## Introduction

Worldwide over 400 million people have diabetes, and the prevalence is increasing rapidly in both developed and developing countries [1]. Previous studies of mostly Western populations have shown that diabetes is typically associated with a 2-fold increased risk of ischaemic heart disease (IHD) [2,3]. Uncertainty remains, however, about whether similar excess risk applies to other populations, and about the strength of the association of diabetes with stroke and, particularly, stroke subtypes [2–4]. Appropriate understanding of these issues is of considerable relevance to China, where stroke rates are high [5].

Since the 1980s, there has been a rapid and substantial increase in the prevalence of diabetes in China [1,6,7], which now affects ~10% of adults [8]. Compared with those in Western populations, individuals with diabetes in China have tended to be leaner and to have worse pancreatic beta-cell function, which may result in greater susceptibility to microvascular complications and cancer than to macrovascular complications [9]. Despite the growing diabetes epidemic, there is limited evidence about the association of diabetes with cardiovascular disease in China [10–14], with previous studies limited by potentially outdated risk estimates [12], highly selected study populations [11], relatively small size [10–12], and lack of proper investigation of the relevance of diabetes duration and other modifiable factors, such as smoking, adiposity, blood pressure, and physical activity, which frequently differ between China and Western populations.

We report relevant findings from a large prospective study, the China Kadoorie Biobank (CKB), of 0.5 million adults, recruited between 25 June 2004 and 15 July 2008 across ten diverse areas of China. The aim of the present study is to examine the association of diabetes—both self-reported and screen-detected—with the risks of major cardiovascular diseases, including IHD and stroke subtypes. By applying population-attributable fractions for cardiovascular mortality from the present study to national cause-specific mortality and representative diabetes prevalence data, we also estimate the number of cardiovascular deaths attributable to diabetes in mainland China.

## Methods

### Ethics Statement

All participants provided written consent prior to participation, including permission for follow-up. Ethics approval was obtained from Oxford University, the Chinese Centre for Disease Control and Prevention (CDC), and the ten study areas’ local CDCs.

### Study Population

Details of the CKB design, methods, and population have been reported previously [15,16]. Briefly, the 2004–2008 baseline survey took place in ten localities across China (five urban and five rural; S1 Fig) chosen to provide diversity in exposure and disease patterns, additionally taking account of logistical considerations, including population stability, death and disease registry quality, and local capacity. All residents aged 35–74 y from 100–150 administrative units (rural villages or urban residential committees) in each area were invited to attend study assessment clinics. Overall, ~30% responded [15], and 512,891 individuals were enrolled (including a few slightly outside the 35–74 y range).

### Data Collection

At study assessment clinics, trained health workers administered laptop-based questionnaires that collected data on socio-demographic status, smoking, alcohol consumption, diet, physical activity (related to leisure, household work, occupation, and commuting [17]), medical history, and—among participants reporting a history of diabetes, IHD, stroke/transient ischaemic attack, and hypertension—current use of medications. Information on age at diagnosis was collected from participants reporting a history of diabetes. Health workers measured height, weight, waist and hip circumference, and blood pressure and took non-fasting venous blood samples (with the time since last food recorded) for storage and immediate on-site testing of plasma glucose level using a SureStep Plus meter (LifeScan). Participants without self-reported diabetes with a plasma glucose level of 7.8–11.0 mmol/l were invited to undergo fasting glucose testing (using the same measurement technique) the following day. Every 4–5 y, a 5%–6% random sample of surviving participants was resurveyed, collating the same data as at baseline, with certain additions.

### Assessment of Diabetes Status

Participants answering “yes” to the question, “Has a doctor ever told you that you had diabetes?” at baseline were defined as having self-reported diabetes; among them, information about age at diagnosis and current medication use was collected. Screen-detected diabetes was defined as not having self-reported diabetes but having a random plasma glucose (RPG) level ≥ 7.0 mmol/l if time since last food ≥ 8 h, RPG level ≥ 11.1 mmol/l if time since last food < 8 h, or fasting plasma glucose level ≥ 7.0 mmol/l on subsequent testing [18]. Participants with screen-detected diabetes were provided with a referral letter and advised to seek formal medical consultation.

### Follow-Up for Mortality and Morbidity

The vital status of each participant was obtained periodically from China CDC’s Disease Surveillance Points, checked annually against local residential and health insurance records and by active confirmation through street committee or village administrators. Causes of death from official death certificates were ICD-10 coded by trained Disease Surveillance Point staff blinded to baseline information. Information on non-fatal outcomes was collected through linkage with established disease surveillance systems (for cancer, IHD, stroke, and diabetes) and, via unique national ID, with the national health insurance system, which records details of ICD-10-coded hospitalisations in all study areas [16].

By 1 January 2014, 25,488 (5.0%) participants had died, and 2,411 (0.5%) were lost to follow-up. For the present study, the primary endpoints were cardiovascular death (ICD-10 I00–I25, I27–I88, I95–I99), myocardial infarction (MI) (ICD-10 I21–I23), major coronary event (MCE) (non-fatal MI or fatal IHD; ICD-10 I20–I25), ischaemic stroke (IS) (ICD-10 I63), intracerebral haemorrhage (ICH) (ICD-10 I61), total stroke (ICD-10 I60, I61, I63, I64), and major occlusive vascular disease (MOVD) (IS or MCE).

### Statistical Analysis

The main analyses excluded participants reporting prior doctor-diagnosed IHD (*n* = 15,472, 3.0%) or stroke/transient ischaemic attack (*n* = 8,884, 1.7%) at baseline. A further 1,081 (0.2%) participants with missing, implausible, or extreme values for blood pressure, height, waist circumference, hip circumference, waist-to-hip ratio, or body mass index (BMI) were excluded, leaving 488,760 individuals (199,896 men, 288,864 women) for the present analyses.

Analyses were done separately for self-reported and screen-detected diabetes, with a common reference group consisting of individuals without self-reported or screen-detected diabetes. The mean values and prevalence of certain variables were calculated by diabetes status, standardised by 5-y age group, sex, and study area. Direct standardisation was used to calculate age-, sex-, and study-area-adjusted disease incidence rates, using the total study population as the standard. Cox regression yielded hazard ratios (HRs) for diabetes versus not, stratified by age at risk (5-y age groups), study area, and, where appropriate, sex, and adjusted for education (no formal education, primary school, middle school, high school, college/university), smoking (never, occasional, ex-regular, current regular), alcohol consumption (never, occasional, ex-regular, reduced, weekly), systolic blood pressure (SBP) (nine groups), and physical activity (five groups). Sensitivity analyses were performed adjusting for age, SBP, and physical activity as continuous variables.

Comparison of HRs for the first four and subsequent years of follow-up revealed no evidence of departure from the proportional hazards assumption. Adjusted HRs were calculated across strata of other cardiovascular risk factors and duration of diabetes at baseline, and chi-square tests for trend and heterogeneity (i.e., effect modification or statistical interaction) were applied to the log HRs and their standard errors [19]. In separate analyses, risk estimates were also adjusted for adiposity. Finally, the HR for MOVD associated with the number of other presenting baseline cardiovascular risk factors (hypertension, overweight or obese, ever regular smoking, physical inactivity) among individuals with diabetes was assessed using the floating absolute risk method [20], which provides estimates of variance across all exposure categories.

Population-attributable risk is *P*(HR − 1)/(1 + *P*[HR − 1]), where *P* is the prevalence of diabetes. By applying age-specific HRs (which can be assumed to approximate the relative risk [21]) to nationally representative, age-specific prevalence of diabetes [8] and national cause-specific mortality data [22], we estimated the number of cardiovascular deaths attributable to diabetes. All analyses used SAS version 9.3.

## Results

Among 488,760 participants without prior cardiovascular disease, the mean baseline age was 51 (standard deviation 11) y, 2.7% (*n* = 13,284) reported a history of doctor-diagnosed diabetes (self-reported diabetes), and a further 2.7% (*n* = 13,051) had screen-detected diabetes, of whom 8,554 (65.5%) were identified through RPG measurement (RPG ≥ 7.0 mmol/l and time since last food ≥ 8 h: *n* = 3,986; RPG ≥ 11.1 mmol/l and time since last food < 8 h: *n* = 4,568), 1,447 (11.1%) through fasting plasma glucose measurement, and 3,050 (23.4%) through both. For both self-reported and screen-detected diabetes, the prevalence was slightly higher in women than in men (2.9% versus 2.5% for self-reported diabetes and 2.8% versus 2.6% for screen-detected diabetes), mainly at ages > 50 y (S2 Fig), and the prevalence was higher in urban than in rural areas (3.9% versus 1.8% for self-reported diabetes and 3.4% versus 2.1% for screen-detected diabetes). Participants reporting a history of diabetes were older and less likely to be current smokers or alcohol drinkers, but more likely to be ex-smokers or ex-drinkers and to be less physically active than those without diabetes, and had an approximately 4-fold greater prevalence of a family history of diabetes (Table 1). Those with screen-detected diabetes were older than and about twice as likely to have a family history of diabetes as those without diabetes, but had comparable smoking and alcohol consumption patterns. The prevalence of overweight or obesity (BMI ≥ 25.0 kg/m^2^) and hypertension (SBP ≥ 140 mm Hg) were higher in participants with diabetes, particularly screen-detected diabetes. Among participants with self-reported diabetes, the median age at diagnosis was 53 (range 4 to 77) y, and the median duration of diabetes at baseline was 4 (range 0 to 56) y, with 76% (*n* = 8,501) reporting use of insulin or oral hypoglycaemic agents.

**Table 1:** Baseline characteristics of participants by diabetes status.

Characteristic	Diabetes			Total (*n* = 488,760)
	None (*n* = 462,425)	Screen-Detected (*n* = 13,051)	Self-Reported (*n* = 13,284)	
**Men** ^a^	189,698 (41.1%)	5,046 (39.1%)	5,152 (41.5%)	199,896 (40.9%)
**Age (years)** ^b^				
30–59	362,567 (78.4%)	8,464 (64.9%)	7,286 (57.3%)	378,317 (77.4%)
60–69	74,308 (16.1%)	3,330 (25.7%)	4,387 (32.1%)	82,025 (16.8%)
70–79	25,550 (5.5%)	1,257 (9.3%)	1,611 (10.6%)	28,418 (5.8%)
Mean (SE)	50.7 (0.0)	55.5 (0.1)	57.7 (0.1)	51.1 (0.0)
**Living in urban area**	195,572 (42.4%)	7,174 (53.3%)	8,236 (58.5%)	210,982 (43.2%)
**Highest level of education ≥6 y** ^c^	228,358 (50.8%)	5,796 (52.6%)	6,258 (48.6%)	240,412 (49.2%)
**Smoking history**				
Never or occasional	312,832 (67.7%)	8,877 (67.2%)	9,354 (68.8%)	331,063 (67.7%)
Ex-regular	24,986 (5.5%)	954 (6.1%)	1,289 (7.7%)	27,229 (5.6%)
Current regular	124,607 (26.8%)	3,220 (26.7%)	2,641 (23.5%)	130,468 (26.7%)
**Alcohol consumption**				
Never	209,524 (45.4%)	6,294 (47.7%)	7,468 (55.3%)	223,286 (45.7%)
Occasional	175,434 (37.8%)	4,350 (34.2%)	4,048 (31.1%)	183,832 (37.6%)
Ex-regular	7,197 (1.6%)	246 (1.9%)	478 (3.9%)	7,921 (1.6%)
Current regular	70,270 (15.2%)	2,161 (16.1%)	1,290 (9.6%)	73,721 (15.1%)
**Physical activity (MET h/d)**				
<10	99,334 (22.0%)	4,315 (25.9%)	5,508 (30.6%)	109,157 (22.3%)
10–14.9	83,887 (18.3%)	2,761 (18.8%)	3,186 (20.9%)	89,834 (18.4%)
≥15.0	279,204 (59.7%)	5,975 (55.3%)	4,590 (48.4%)	289,769 (59.3%)
Mean (SE)	21.6 (0.0)	20.3 (0.1)	18.1 (0.2)	21.5 (0.0)
**SBP (mm Hg)**				
<120	154,757 (33.1%)	2,001 (18.4%)	2,186 (22.8%)	158,944 (32.5%)
120–139	183,855 (39.7%)	4,731 (39.2%)	4,695 (37.6%)	193,281 (39.6%)
≥140	123,813 (27.2%)	6,319 (42.4%)	6,403 (39.6%)	136,535 (27.9%)
Mean (SE)	130.2 (0.0)	138.6 (0.2)	136.4 (0.3)	130.6 (0.0)
**BMI (kg/m** ^**2**^)				
<22.0	158,040 (34.0%)	2,443 (19.6%)	2,792 (23.9%)	163,275 (33.4%)
22.0 to <25.0	159,581 (34.5%)	3,917 (29.9%)	4,528 (34.5%)	168,026 (34.4%)
≥25.0	144,804 (31.6%)	6,691 (50.5%)	5,964 (41.6%)	157,459 (32.2%)
Mean (SE)	23.5 (0.0)	25.1 (0.0)	24.5 (0.1)	23.6 (0.0)
**Family history of diabetes** ^d^ ^,^ ^e^	29,310 (6.7%)	1,616 (13.9%)	3,192 (26.5%)	34,118 (7.3%)
**Mean (SE) RPG (mmol/l)** ^f^	5.7 (0.0)	13.3 (0.1)	11.8 (0.0)	6.0 (0.0)
**Self-reported medication use**				
Insulin^g^			1,436 (12.8%)	
Oral hypoglycaemic agent^g^			7,347 (65.6%)	
CVD medication^h^ ^,^ ^i^			1,919 (17.0%)	
Aspirin^h^			248 (2.1%)	
Statin^h^			107 (1.0%)	
Anti-hypertensive^h^ ^,^ ^j^			1,743 (15.5%)	

Data are given as *n* (percent) or mean (standard error). Standardised to the age, sex, and study area structure of the study population. *p*-Values for differences between participants with self-reported, screen-detected, and no diabetes: all <0.001.

^a^Standardised to age and study area structure only.

^b^Standardised to sex and study area structure only.

^c^Highest level of education middle school, high school, or college/university.

^d^First-degree relatives.

^e^Data missing for 23,368 participants.

^f^Data missing for 8,125 participants.

^g^Data missing for 2,091 participants.

^h^Data missing for 1,735 participants.

^i^Statin, aspirin, ACE inhibitor, beta-blocker, diuretic, calcium channel antagonist.

^j^ACE inhibitor, beta-blocker, diuretic, calcium channel antagonist.

CVD, cardiovascular disease; MET, metabolic equivalent of task; SE, standard error.

### Self-Reported Diabetes and Cardiovascular Disease Risk

During ~3.4 million person-years (mean 7 y per person) of follow-up, 7,353 (1.5%) participants died from cardiovascular disease. Individuals with self-reported diabetes had a 2-fold increased hazard of cardiovascular mortality (HR 2.07, 95% CI 1.90–2.26) (Table 2), and the HR for cardiovascular mortality was greater in women than in men (2.37, 95% CI 2.11–2.65, versus 1.73, 95% CI 1.50–2.00) (S3 Fig). Likewise, there were significantly increased risks of MOVD (1.77, 1.69–1.86), MCE (2.44, 2.18–2.73), and IS (1.68, 1.60–1.77) among those with self-reported diabetes (Table 2). For IHD and IS, the HRs were somewhat greater for fatal than non-fatal events. For IS and MOVD (S4 Fig), the associations were stronger at younger ages (both *p* for trend < 0.001) and in rural areas (*p* for heterogeneity = 0.003 and < 0.001, respectively). The risk of MCE varied little across population subgroups.

**Table 2:** Adjusted hazard ratios for incident cardiovascular diseases by diabetes status.

Diabetes Group	Outcome	Diabetes		No Diabetes		Model A^b^		Model B^c^	
		Number of Events	Rate per 100,000 Person-Years^a^	Number of Events	Rate per 100,000 Person-Years^a^	HR	95% CI	HR	95% CI
**Self-reported diabetes** ^d^	**CVD mortality**	571	351.1	6,345	133.5	2.49	2.28–2.72	2.07	1.90–2.26
	**MOVD**	1,938	1,177.8	20,876	536.8	2.03	1.94–2.13	1.77	1.69–1.86
	**IHD**								
	Fatal MI	127	88.1	1,237	25.7	3.03	2.51–3.65	2.67	2.21–3.23
	Non-fatal MI	122	78.2	1,087	27.3	2.52	2.08–3.06	2.18	1.80–2.65
	MCE	368	211.9	3,097	69.2	2.80	2.50–3.13	2.44	2.18–2.73
	**Stroke—IS**								
	Fatal	43	24.5	416	8.6	2.65	1.92–3.66	2.20	1.59–3.04
	Non-fatal	1,589	952.6	17,730	463.6	1.91	1.81–2.01	1.67	1.59–1.76
	Any	1,632	977.2	18,146	472.1	1.92	1.82–2.02	1.68	1.60–1.77
	**Stroke—ICH**								
	Fatal	100	109.6	1,891	42.6	2.01	1.64–2.47	1.59	1.30–1.96
	Non-fatal	92	66.3	2,340	61.8	1.22	0.99–1.51	1.00	0.81–1.23
	Any	192	175.9	4,231	104.4	1.54	1.33–1.78	1.24	1.07–1.44
	**Total stroke**								
	Fatal	168	154.8	2,575	56.9	2.20	1.88–2.58	1.78	1.52–2.09
	Non-fatal	1,749	1,068.6	21,157	553.5	1.84	1.75–1.94	1.60	1.52–1.68
	Any	1,917	1,223.3	23,732	610.4	1.87	1.78–1.96	1.61	1.54–1.69
**Screen-detected diabetes** ^d^	**CVD mortality**	437	294.0	6,345	133.5	1.98	1.80–2.18	1.66	1.51–1.83
	**MOVD**	1,356	1,021.1	20,876	536.8	1.73	1.64–1.83	1.50	1.41–1.58
	**IHD**								
	Fatal MI	81	60.6	1,237	25.7	1.90	1.52–2.39	1.68	1.34–2.12
	Non-fatal MI	68	52.3	1,087	27.3	1.70	1.33–2.18	1.51	1.18–1.93
	MCE	213	140.0	3,097	69.2	1.84	1.60–2.11	1.62	1.40–1.86
	**Stroke—IS**								
	Fatal	33	19.3	416	8.6	2.19	1.53–3.13	1.85	1.29–2.64
	Non-fatal	1,144	869.5	17,730	463.6	1.70	1.60–1.81	1.47	1.39–1.57
	Any	1,177	888.8	18,146	472.1	1.71	1.62–1.82	1.48	1.40–1.57
	**Stroke—ICH**								
	Fatal	116	96.8	1,891	42.6	2.02	1.67–2.44	1.62	1.34–1.96
	Non-fatal	73	69.1	2,340	61.8	1.05	0.83–1.32	0.81	0.64–1.03
	Any	189	165.9	4,231	104.4	1.49	1.28–1.72	1.17	1.01–1.36
	**Total stroke**								
	Fatal	168	129.1	2,575	56.9	2.05	1.75–2.40	1.66	1.42–1.95
	Non-fatal	1,268	981.7	21,157	553.5	1.63	1.54–1.73	1.39	1.32–1.48
	Any	1,436	1,110.8	23,732	610.4	1.67	1.59–1.77	1.42	1.34–1.49

Events classified as fatal if death from any cause within 28 d.

^a^Age-, sex-, and study-area-standardised rates.

^b^Model A: stratified by age, sex, and study area.

^c^Model B: additionally adjusted for education, smoking, alcohol consumption, physical activity, and SBP.

^d^Reference group is individuals without self-reported or screen-detected diabetes.

CVD, cardiovascular disease.

Self-reported diabetes was also associated with significant, but more modest, excess risk of ICH, with an adjusted HR of 1.24 (95% CI 1.07–1.44), more extreme for fatal (1.59, 95% CI 1.30–1.96) than for non-fatal (1.00, 95% CI 0.81–1.23) events (Table 2). The risk varied little across participant subgroups (S5 Fig). For total stroke, self-reported diabetes was associated with an adjusted HR of 1.61 (95% CI 1.54–1.69; Table 2).

Additional adjustment for waist-to-hip ratio modestly, but non-significantly, attenuated the HRs for ischaemic cardiovascular diseases, but not for ICH or cardiovascular mortality (S1 Table). In sensitivity analyses excluding participants who developed incident diabetes during follow-up (*n* = 8,896), HRs for all cardiovascular diseases remained largely unchanged (S2 Table). Sensitivity analyses adjusting for age, SBP, and physical activity as continuous variables also produced similar findings.

### Screen-Detected Diabetes and Cardiovascular Disease Risk

Among participants with screen-detected diabetes, the excess risks were also highly significant, though somewhat less extreme compared with self-reported diabetes. For cardiovascular mortality, the adjusted HR was 1.66 (95% CI 1.51–1.83), while for MOVD, MCE, and IS the adjusted HRs were 1.50 (95% CI 1.41–1.58), 1.62 (95% CI 1.40–1.86), and 1.48 (95% CI 1.40–1.57), respectively. Similarly, for ICH the excess risk was more modest (1.17, 95% CI 1.01–1.36) (Table 2). There was little evidence of heterogeneity in the observed HRs for MCE, ICH (S6 Fig), or cardiovascular mortality (S7 Fig) across population subgroups. For IS and MOVD (S8 Fig), however, the risk was somewhat greater at younger ages (both *p* for trend < 0.001), in rural residents (*p* for heterogeneity < 0.001 and 0.003, respectively), and in more physically active individuals (*p* for trend 0.008 and 0.006, respectively).

**Fig 1:**
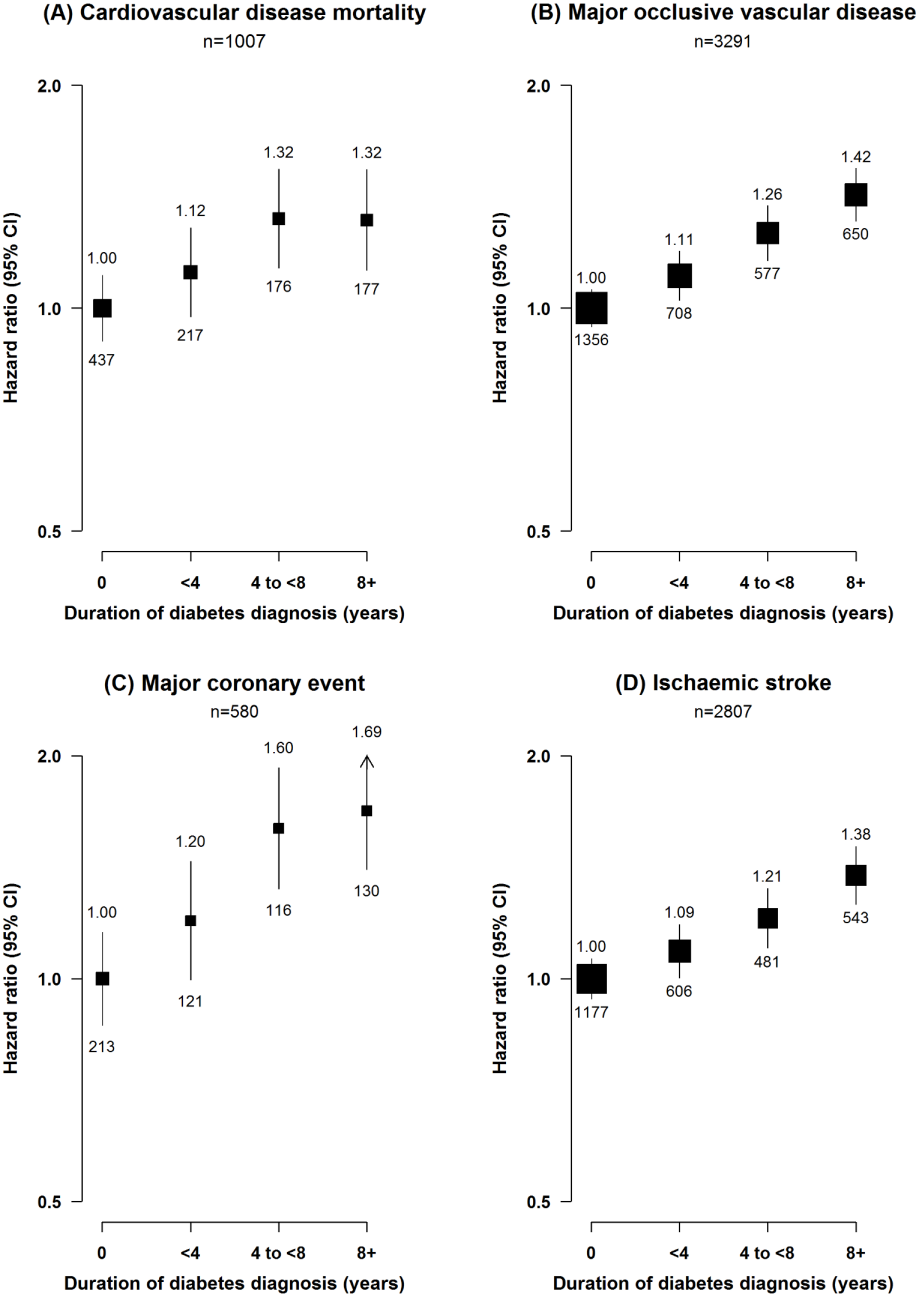
Adjusted hazard ratios for cardiovascular diseases by duration of diabetes diagnosis. (A) Cardiovascular disease mortality; (B) MOVD; (C) MCE; (D) IS. Stratified by age, sex, and study area and adjusted for education, smoking, alcohol consumption, physical activity, and SBP. Diabetes duration defined as 0 y in individuals with screen-detected diabetes. Diabetes duration data missing or implausible for 17 participants. Squares represent the HR, with area inversely proportional to the variance of the log HR. Vertical lines represent the corresponding 95% confidence intervals. Numbers above the CIs are HRs, and numbers below the CIs are the number of participants with cardiovascular events.

**Fig 2:**
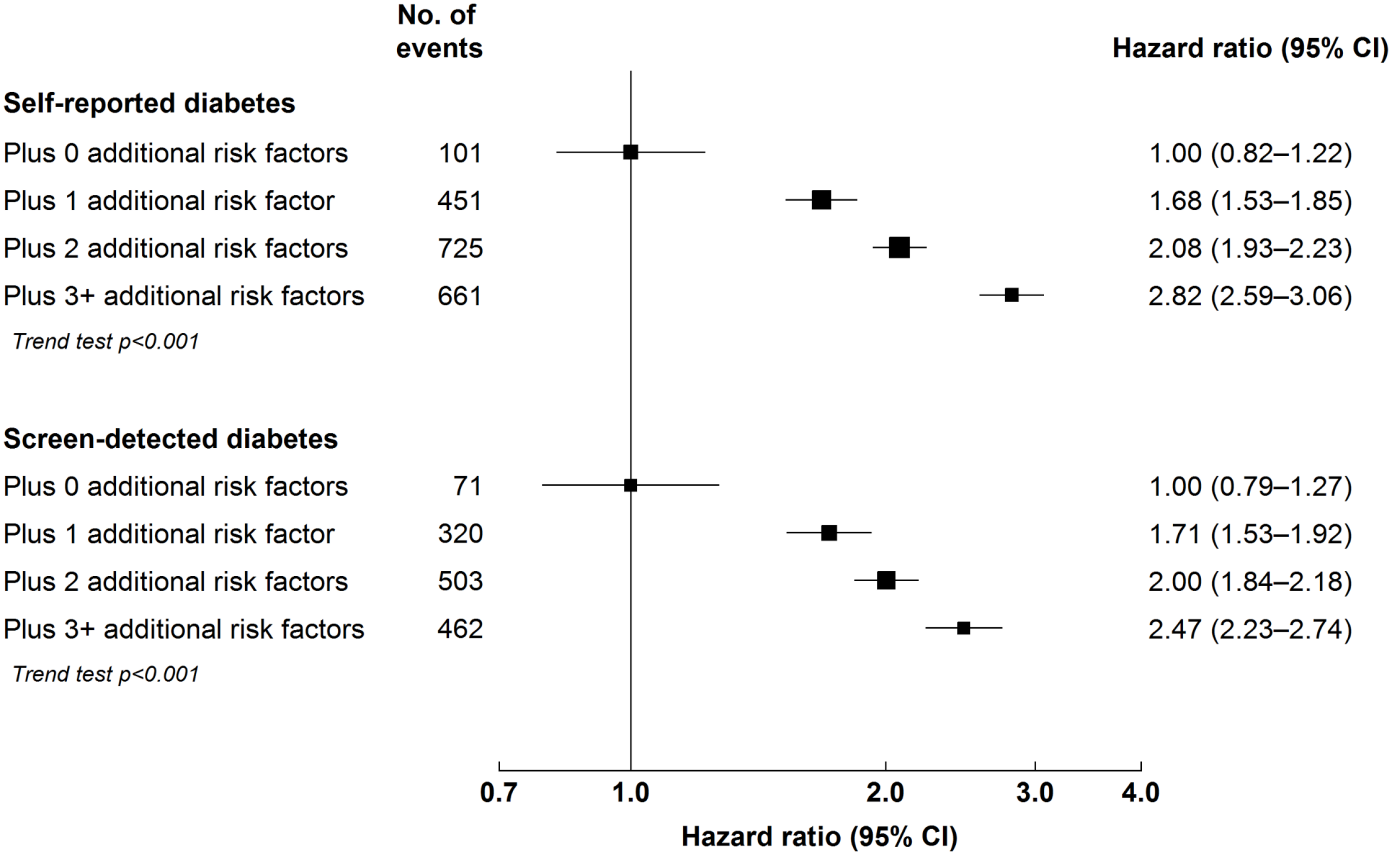
Adjusted hazard ratios for major occlusive vascular disease by number of cardiovascular risk factors at baseline among individuals with diabetes. Stratified by age, sex, and study area and adjusted for education and alcohol consumption. Cardiovascular risk factors: hypertension (self-reported hypertension, mean SBP ≥ 140 mm Hg or diastolic blood pressure ≥ 90 mm Hg), overweight or obesity (BMI ≥ 25 kg/m^2^), ever regular smoking, and physical inactivity (<10 metabolic equivalent of task hours/day). Squares represent the HR, with area inversely proportional to the variance of the log HR. Horizontal lines represent the corresponding 95% confidence intervals.

Among individuals with diabetes, the risk of major ischaemic cardiovascular events increased progressively with longer duration of diabetes (*p* for trend < 0.001; Fig 1), but no such trend was seen for ICH (*p* = 0.3). Similarly, among individuals with self-reported diabetes, the risk of MOVD increased progressively with the number of other presenting cardiovascular risk factors, with adjusted HRs of 1.68 (95% CI 1.53–1.85), 2.08 (95% CI 1.93–2.23), and 2.82 (95% CI 2.59–3.06) in those with one, two, and three or more risk factors, respectively, compared with participants with self-reported diabetes who had no other cardiovascular risk factors (*p* for trend < 0.001; Fig 2). A similar gradient was seen for screen-detected diabetes (*p* for trend < 0.001; Fig 2).

### Diabetes-Attributable Cardiovascular Mortality

By applying the age-specific population-attributable fractions for cardiovascular mortality in the present study to diabetes prevalence derived from a recent nationally representative survey [8] and the number of sex- and age-specific cardiovascular deaths in mainland China [22], it was estimated that 489,676 (95% CI 335,777–681,202) cardiovascular deaths could be attributable to diabetes in 2010.

## Discussion

Our study provides, to our knowledge, the first large-scale prospective evidence of the cardiovascular consequences of diabetes among adults in China. It showed that self-reported doctor-diagnosed diabetes was associated with 1.5- to 2.5-fold increased risks of cardiovascular mortality and incident IHD and IS. Moreover, it provided strong prospective evidence of a significant, though more modest, adverse association of diabetes with ICH risk. Individuals with screen-detected diabetes also had significantly increased cardiovascular disease risks. If these associations are causal, then almost 0.5 million cardiovascular deaths a year in China can now be attributed to diabetes.

Previous studies of predominantly Western populations have shown approximate doubling of IHD risk with diabetes [2,3,10,11,23]. The comparability of our risk estimates with previous studies suggests that differences between East Asian and Western populations in the relative importance of insulin resistance and beta-cell dysfunction in the aetiology of diabetes [9] have little, if any, impact on associated IHD risk. The method of ascertainment of IHD in the CKB may misclassify a proportion of individuals with “silent” IHD, which is more prevalent among individuals with diabetes [24], leading to underestimation of diabetes-associated IHD risk. The more modest effects of screen-detected—compared to self-reported—diabetes may reflect the shorter duration of, or less severe glycaemic aberration in, screen-detected diabetes, and greater potential for misclassification.

The CKB provides reliable evidence about the association of diabetes with stroke and, in particular, stroke subtypes. The association of screen-detected diabetes with IS in our study is largely consistent with data from studies of mostly Western populations [3]; the association with self-reported diabetes was moderately weaker than in those studies [2,3] and also weaker than in a small prospective Chinese study of 30,000 men and women [10] and a study—based on routine data and adjusted only for age—in Taiwan [25]. In mainland China, computed tomography or MRI is widely used, often leading to detection of a relatively high proportion of lacunar infarcts without major, or any, apparent focal neurological deficit [26]. The relatively low IS case fatality rate in our study might explain, at least in part, the more modest association with diabetes.

Existing evidence relating ICH to diabetes is more limited [2,3,10,25,27], partly due to lower ICH rates in Western populations [2,3] and the lack of widespread imaging in earlier studies. Where available, findings have been inconsistent [2–4,10,25]. Two of the largest studies in Western populations, including 1,000 [3] and 2,500 [4] ICH events, found modest elevated risks associated with known diabetes (HR 1.56 [3] and 1.28 [4]). Our study included almost 5,000 well-characterised ICH cases and provides the most reliable evidence to date of a positive, but modest, association between self-reported diabetes and ICH, and the first opportunity, to our knowledge, to investigate effect modification beyond age and sex. The large number of well-characterised stroke events (~90% of validated stroke events were confirmed with computed tomography/MRI) is a strength of the CKB. Outcome adjudication, through review of medical records for all IHD and stroke cases, is currently underway; findings to date have shown high positive predictive values for IHD events (~85%) and for stroke (~90%). The apparent difference in excess risk between fatal and non-fatal cardiovascular disease events could reflect poorer survival following such events in individuals with diabetes [28,29] or more severe disease in fatal cases.

The large urban—rural differences in excess risk associated with self-reported diabetes in the CKB, especially for IS, may reflect the higher proportion of undiagnosed diabetes cases in rural areas, such that self-reported diabetes in rural areas may represent more severe disease. More frequent use of diabetes medications in rural areas supports this hypothesis. Several studies have previously reported greater diabetes-associated IHD risk [3,23] and, less consistently, stroke risk [3,4,30] in women. We found a sex difference for cardiovascular mortality only, and this may have contributed to the higher diabetes-associated risks in never or occasional smokers and never or occasional alcohol drinkers. Diabetes, particularly type 2 diabetes, frequently coexists with other cardiovascular risk factors [31]. In our study population, the cardiovascular disease risk among those with diabetes was also dependent on the presence of other common and modifiable cardiovascular risk factors, which highlights the need for multi-factorial approaches to managing cardiovascular disease risk, even after development of diabetes. The small proportion of participants with self-reported diabetes who reported use of medications for lowering cardiovascular disease risk suggests that there is considerable scope for improvement in this regard in China. The impact of diabetes diagnosis duration on disease risk is largely consistent with the almost 2-fold greater odds of prevalent cardiovascular disease in early-onset (<40 y of age), compared to late-onset (≥40 y), diabetes in China, with the majority of the association explained by diabetes duration [32].

The CKB was not designed to be nationally representative, but given the size and diversity of the CKB study population and the CKB’s minimal loss to follow-up, this would not be expected to bias risk estimates or reduce their generalisability to the Chinese adult population [33]. Furthermore, the prevalence of diabetes in the CKB was similar to that reported in a reasonably contemporaneous representative Chinese survey in 2000–2001 [7]. More recent surveys have reported a higher rate of diabetes in China [7], with, for example, a prevalence rate of 11.6% in the 2010 survey [8]. As well as reflecting secular trends, the lower prevalence of diabetes in the CKB may reflect different study settings, sampling methods, and approaches used to identify undiagnosed diabetes. The prevalence of self-reported diabetes was, however, similar (3.5% in the 2010 survey versus 2.7% in the present CKB population and 3.2% in the CKB population including individuals with prior cardiovascular disease). Although self-reported diabetes is widely used [33], it may be subject to misclassification. However, amongst almost 30,000 participants resurveyed in the CKB, over 90% of those who reported a history of diabetes at baseline gave the same answer at resurvey. Furthermore, the mean plasma glucose level among those with self-reported diabetes was consistent with the diagnosis. We did not collect specific information on diabetes type, but, based on age at diagnosis and medication use, <1% of diabetes in the CKB would likely be type 1.

Separate examination of screen-detected diabetes should reduce bias from lifestyle changes and treatment following diagnosis, although screen-detected diabetes may be subject to greater misclassification resulting from use of RPG [34] measurement on a glucometer [35] for diagnosis. Such misclassification likely contributed to the lower screen-detected diabetes prevalence in the CKB compared to the most recent national survey [8] (2.7% in CKB versus 8.1% in the 2010 survey), which used multiple glycaemic indicators (oral glucose tolerance testing, fasting plasma glucose, and HbA1c), and would result in underestimates of diabetes-associated risks. However, excluding participants who developed new diabetes during follow-up (identified from mortality, disease surveillance, and national health insurance data) did not materially alter risk estimates. The high proportion of undiagnosed diabetes [8] and the elevated cardiovascular risk associated with it lend support to population screening for diabetes to enable more effective primary prevention of cardiovascular diseases. Data on lipids are not currently available; this may have resulted in residual confounding, although progressive adjustment for confounders in one published study suggests that this confounding would be minimal [3]. Similarly, renal function data are not currently available in the CKB; this precludes investigation of effect modification of the association of diabetes with cardiovascular diseases but would not bias our risk estimates. Additional adjustment of presented risk estimates for other potential confounding variables, including dietary intake, had minimal effect.

In China, about 10% of the adult population is estimated to have diabetes [8]. The present nationwide prospective study provides large-scale evidence from mainland China that individuals with diabetes are at significantly increased risk of major cardiovascular diseases, similar in magnitude to that observed in Western populations [2,3]. Much of the diabetes-associated excess risk is likely to be causal, accounting for almost 0.5 million cardiovascular deaths annually in China. With further adverse changes in lifestyle, the prevalence of diabetes will likely increase further in China, especially among young adults [1,7], foreshadowing an even greater diabetes-attributable burden of cardiovascular (and other) diseases.

## Supporting Information

S1 Fig Locations of the China Kadoorie Biobank recruitment centres. (PDF)

S2 Fig Age- and sex-specific prevalence of diabetes. (PDF)

S3 Fig Adjusted hazard ratios for cardiovascular disease mortality by self-reported diabetes status. (PDF)

S4 Fig Adjusted hazard ratios for major occlusive vascular disease by self-reported diabetes status. (PDF)

S5 Fig Adjusted hazard ratios for intracerebral haemorrhage by self-reported diabetes status. (PDF)

S6 Fig Adjusted hazard ratios for intracerebral haemorrhage by screen-detected diabetes status. (PDF)

S7 Fig Adjusted hazard ratios for cardiovascular disease mortality by screen-detected diabetes status. (PDF)

S8 Fig Adjusted hazard ratios for major occlusive vascular disease by screen-detected diabetes status. (PDF)

S1 Table Adjusted hazard ratios for incident cardiovascular diseases by diabetes status, additionally adjusted for adiposity. (PDF)

S2 Table Adjusted hazard ratios for incident cardiovascular diseases by self-reported diabetes status excluding participants who developed incident diabetes during follow-up. (PDF)

S3 Table Adjusted hazard ratios for incident cardiovascular diseases by diabetes status. (PDF)

S1 Text Original analysis plan and modifications for the presented analyses. (DOCX)

S2 Text STROBE Checklist. (DOC)

## Abbreviations

BMI: body mass index
CDC: Centre for Disease Control and Prevention
CKB: China Kadoorie Biobank
HR: hazard ratio
ICH: intracerebral haemorrhage
IHD: ischaemic heart disease
IS: ischaemic stroke
MCE: major coronary event
MI: myocardial infarction
MOVD: major occlusive vascular disease
RPG: random plasma glucose
SBP: systolic blood pressure
