# Supplementary figures and images for: Risks and Population Burden of Cardiovascular Diseases Associated with Diabetes in China: A Prospective Study of 0.5 Million Adults
Source: PLoS Med. 2016 Jul 5;13(7):e1002026. doi: 10.1371/journal.pmed.1002026 (PMC4933372; doi:10.1371/journal.pmed.1002026)

**S1 Fig. Locations of the China Kadoorie Biobank recruitment centres**

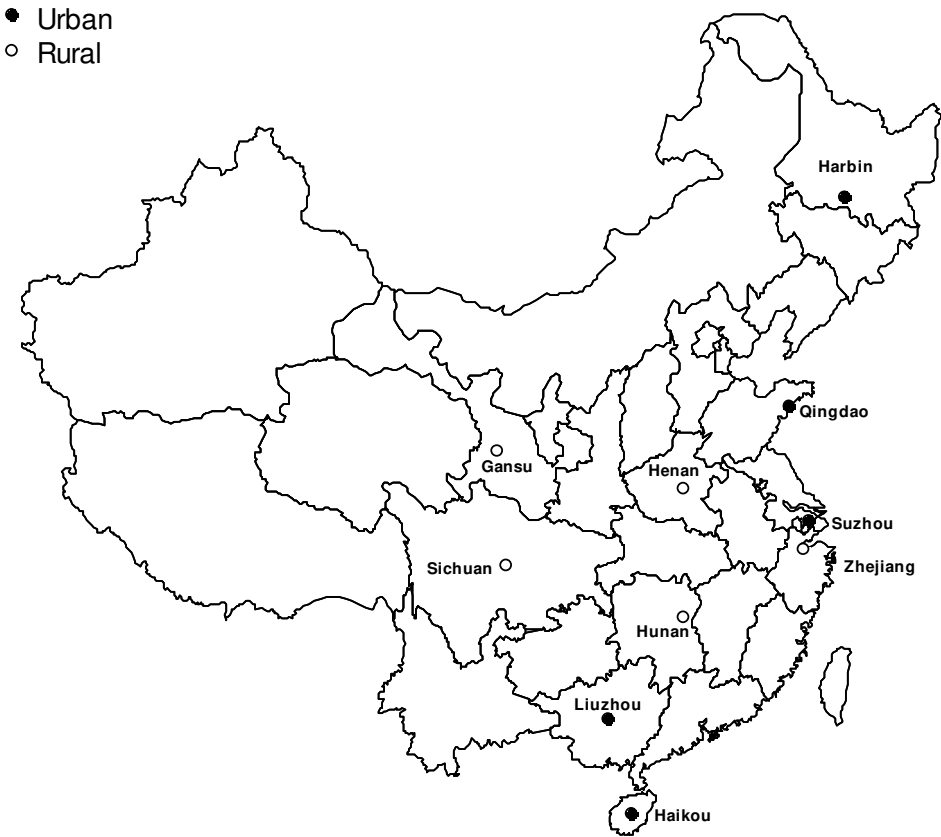

Supplement: S1 Fig — (PDF) [file pmed.1002026.s001.pdf]

**S2 Fig. Age- and sex-specific prevalence of diabetes**

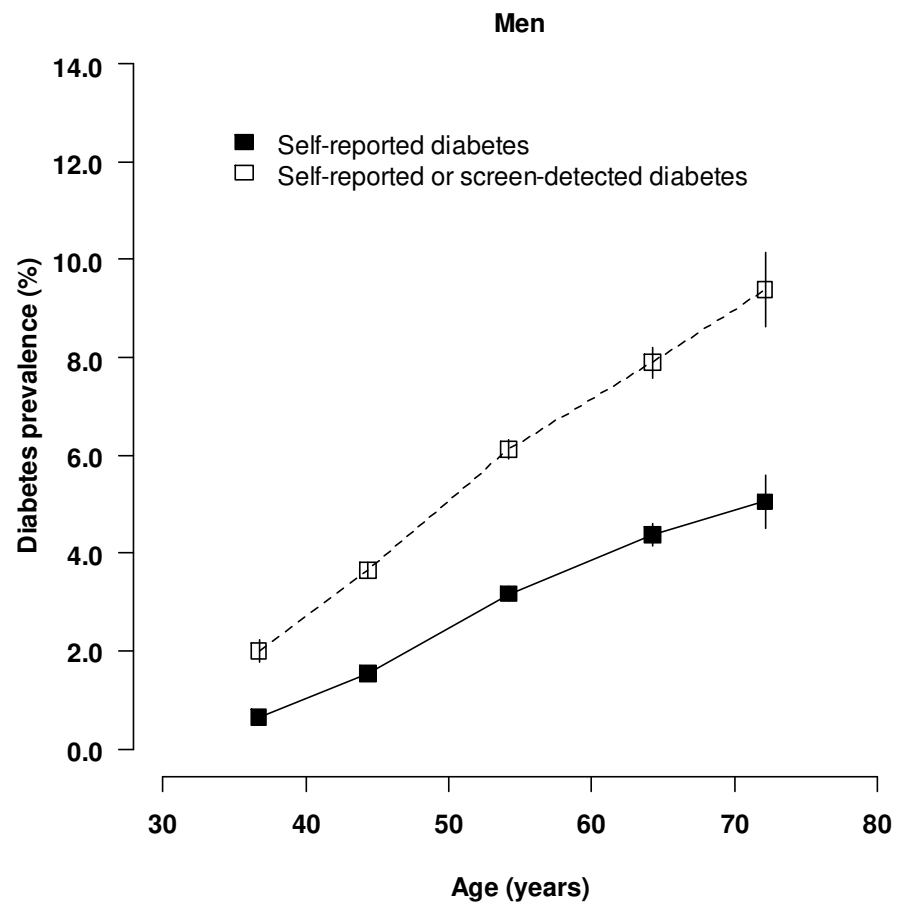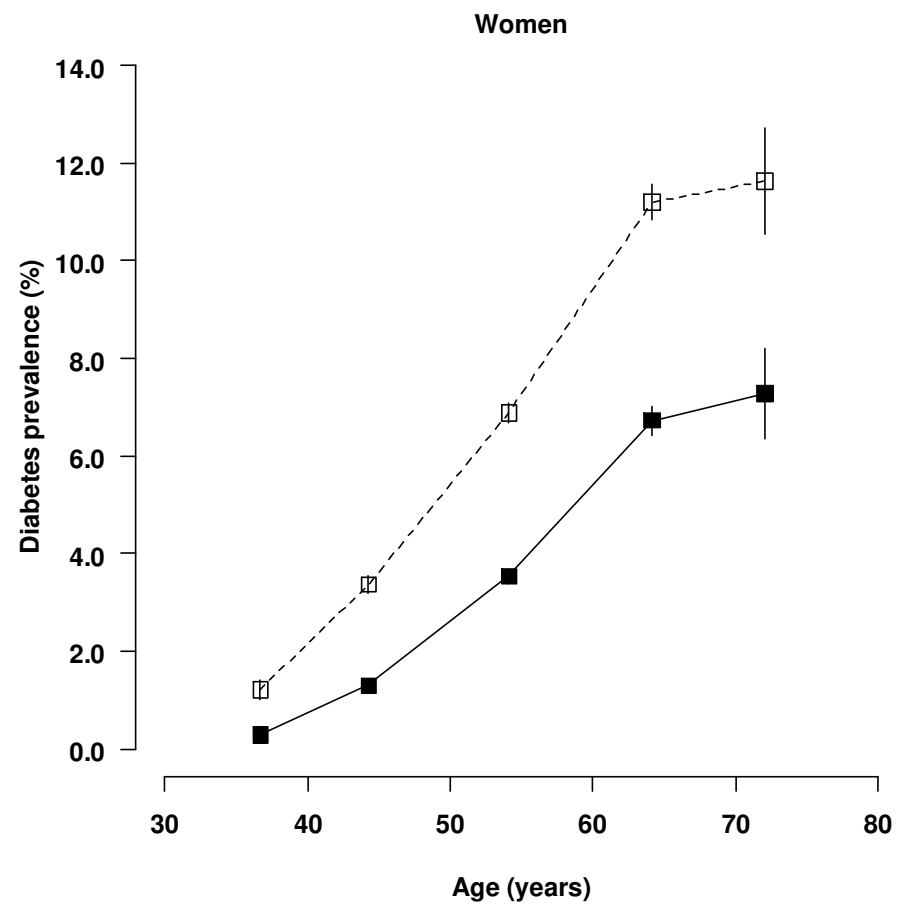

Supplement: S2 Fig — (PDF) [file pmed.1002026.s002.pdf]

**S5 Fig. Adjusted hazard ratios for intracerebral haemorrhage by self-reported diabetes status**

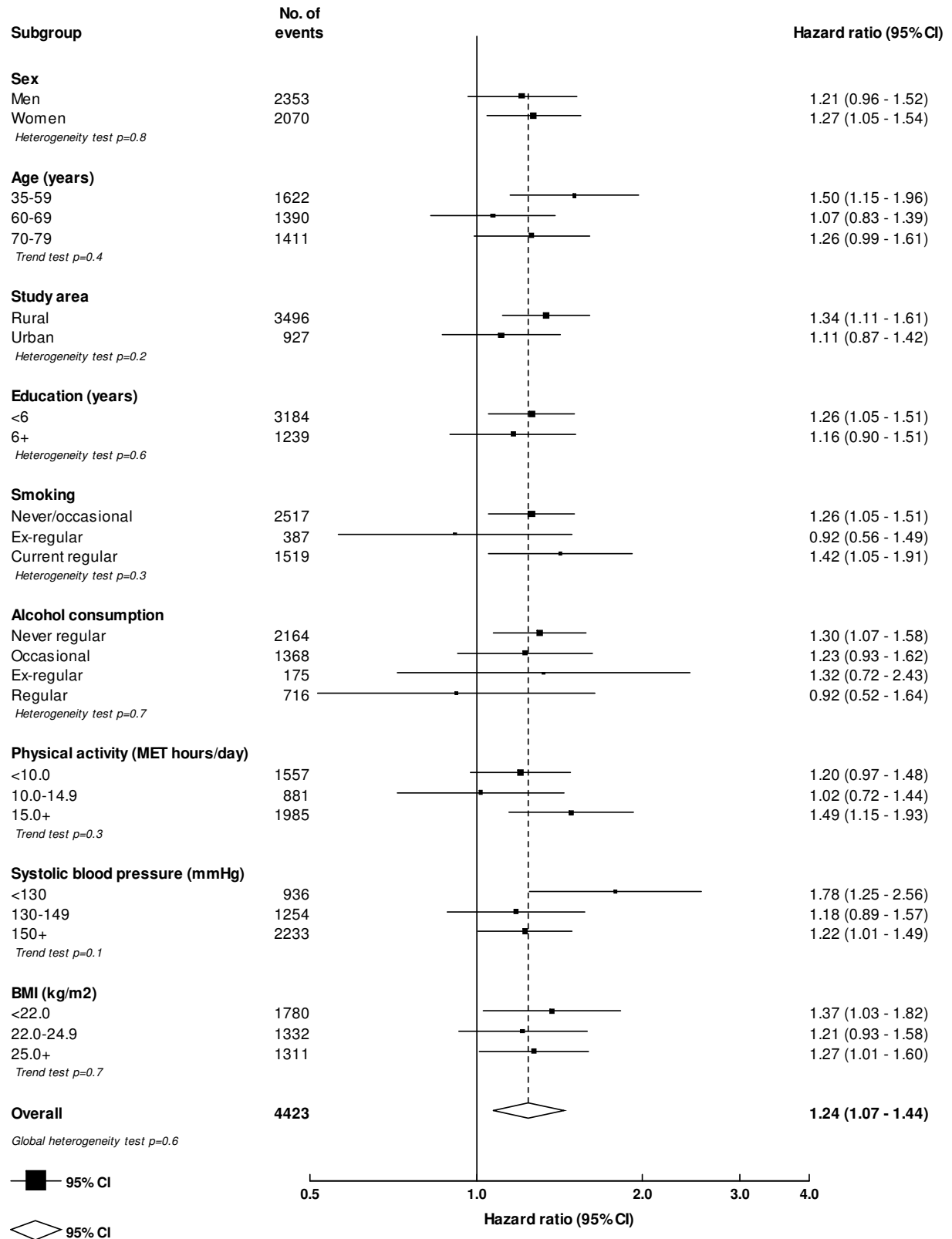

Supplement: S5 Fig — (PDF) [file pmed.1002026.s005.pdf]
