# Supplementary material for: Risks and Population Burden of Cardiovascular Diseases Associated with Diabetes in China: A Prospective Study of 0.5 Million Adults
Source: PLoS Med. 2016 Jul 5;13(7):e1002026. doi: 10.1371/journal.pmed.1002026 (PMC4933372; doi:10.1371/journal.pmed.1002026)

S3 Fig. Adjusted hazard ratios for cardiovascular disease mortality by self-reported diabetes status

Subgroup	No. of events	Hazard ratio (95% CI)
**Sex**		
Men	3861	1.73 (1.50 - 2.00)
Women	3055	2.37 (2.11 - 2.65)
Heterogeneity test p<0.001		
**Age (years)**		
35-59	1525	2.54 (2.06 - 3.13)
60-69	2042	2.03 (1.73 - 2.37)
70-79	3349	1.99 (1.76 - 2.25)
Trend test p=0.08		
**Study area**		
Rural	5077	2.05 (1.82 - 2.32)
Urban	1839	2.11 (1.86 - 2.41)
Heterogeneity test p=0.7		
**Education (years)**		
<6	5212	2.06 (1.85 - 2.30)
6+	1704	2.02 (1.72 - 2.37)
Heterogeneity test p=0.8		
**Smoking**		
Never/occasional	3640	2.23 (2.00 - 2.49)
Ex-regular	751	1.52 (1.16 - 1.98)
Current regular	2525	2.03 (1.69 - 2.43)
Heterogeneity test p=0.03		
**Alcohol consumption**		
Never regular	3418	2.29 (2.04 - 2.57)
Occasional	2131	2.01 (1.70 - 2.38)
Ex-regular	334	1.27 (0.83 - 1.93)
Regular	1033	1.57 (1.11 - 2.22)
Heterogeneity test p=0.01		
**Physical activity (MET hours/day)**		
<10.0	3226	1.96 (1.74 - 2.20)
10.0-14.9	1270	2.08 (1.69 - 2.56)
15.0+	2420	2.45 (2.04 - 2.94)
Trend test p=0.05		
**Systolic blood pressure (mmHg)**		
<130	1618	2.29 (1.84 - 2.84)
130-149	1940	2.18 (1.85 - 2.57)
150+	3358	2.05 (1.82 - 2.31)
Trend test p=0.4		
**BMI (kg/m2)**		
<22.0	2932	2.10 (1.77 - 2.50)
22.0-24.9	2000	1.96 (1.67 - 2.31)
25.0+	1984	2.23 (1.95 - 2.56)
Trend test p=0.5		
**Overall**	**6916**	**2.07 (1.90 - 2.26)**

Global heterogeneity test p=0.009

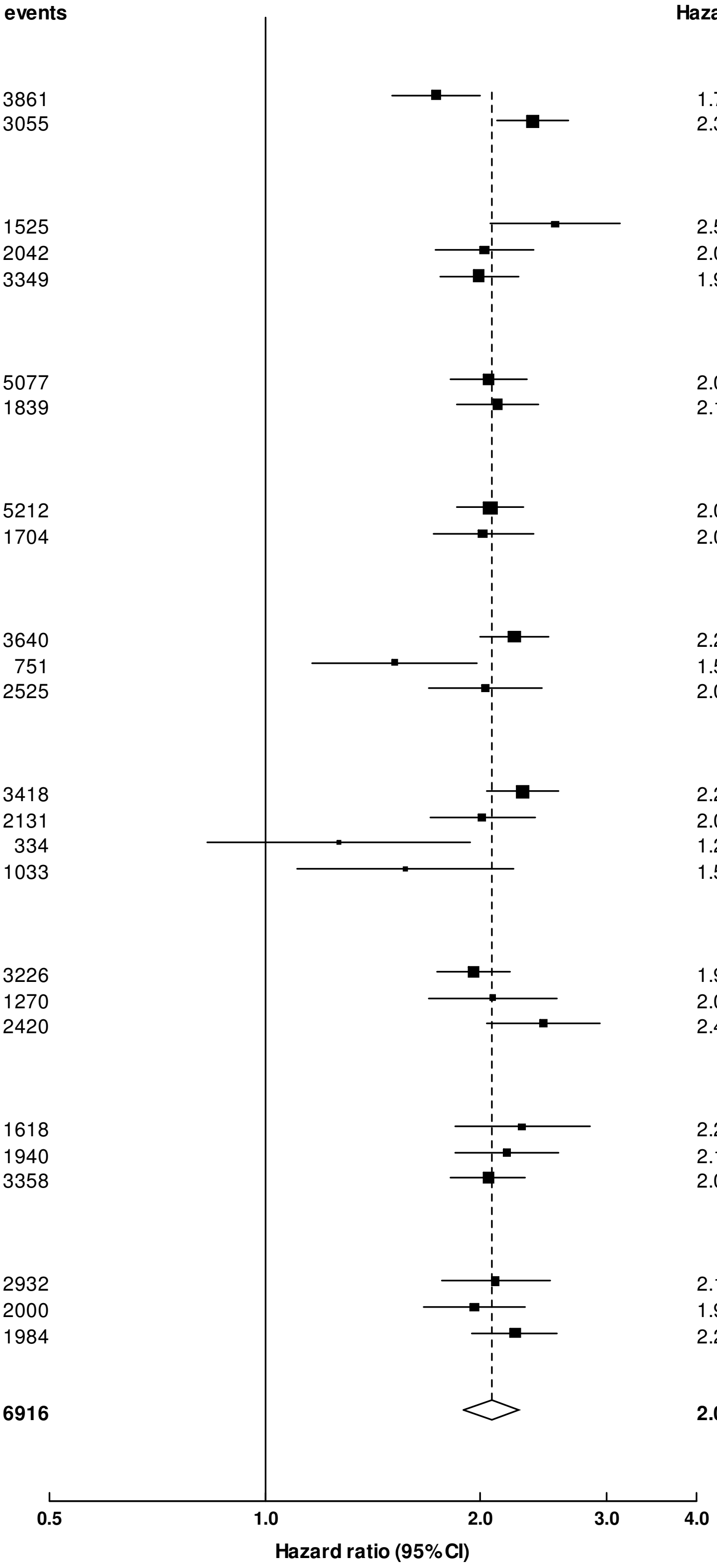

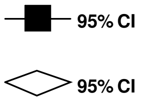

Supplement: S3 Fig — (PDF) [file pmed.1002026.s003.pdf]
