# Supplementary material for: Risks and Population Burden of Cardiovascular Diseases Associated with Diabetes in China: A Prospective Study of 0.5 Million Adults
Source: PLoS Med. 2016 Jul 5;13(7):e1002026. doi: 10.1371/journal.pmed.1002026 (PMC4933372; doi:10.1371/journal.pmed.1002026)
Supplement: S4 Fig — (PDF) [file pmed.1002026.s004.pdf]

S4 Fig. Adjusted hazard ratios for major occlusive vascular disease by self-reported diabetes status

Subgroup	No. of events	Hazard ratio (95% CI)
**Sex**		
Men	10765	1.76 (1.64 - 1.90)
Women	12049	1.80 (1.69 - 1.91)
Heterogeneity test p=0.7		
**Age (years)**		
35-59	7669	2.09 (1.89 - 2.30)
60-69	7567	1.80 (1.67 - 1.95)
70-79	7578	1.61 (1.49 - 1.74)
Trend test p<0.001		
**Study area**		
Rural	11024	2.03 (1.87 - 2.21)
Urban	11790	1.69 (1.60 - 1.79)
Heterogeneity test p<0.001		
**Education (years)**		
<6	12555	1.85 (1.73 - 1.98)
6+	10259	1.68 (1.57 - 1.80)
Heterogeneity test p=0.06		
**Smoking**		
Never/occasional	14137	1.78 (1.68 - 1.88)
Ex-regular	2039	1.75 (1.52 - 2.01)
Current regular	6638	1.83 (1.65 - 2.04)
Heterogeneity test p=0.8		
**Alcohol consumption**		
Never regular	10249	1.75 (1.63 - 1.87)
Occasional	8521	1.88 (1.73 - 2.04)
Ex-regular	639	1.61 (1.25 - 2.08)
Regular	3405	1.80 (1.54 - 2.10)
Heterogeneity test p=0.5		
**Physical activity (MET hours/day)**		
<10.0	9485	1.81 (1.69 - 1.93)
10.0-14.9	5156	1.68 (1.52 - 1.85)
15.0+	8173	1.84 (1.67 - 2.03)
Trend test p=1		
**Systolic blood pressure (mmHg)**		
<130	7576	1.89 (1.71 - 2.09)
130-149	7200	1.91 (1.76 - 2.07)
150+	8038	1.73 (1.61 - 1.86)
Trend test p=0.1		
**BMI (kg/m2)**		
<22.0	6421	1.79 (1.60 - 2.00)
22.0-24.9	7350	1.78 (1.64 - 1.94)
25.0+	9043	1.74 (1.62 - 1.86)
Trend test p=0.6		
**Overall**	**22814**	**1.77 (1.69 - 1.86)**

Global heterogeneity test p<0.001

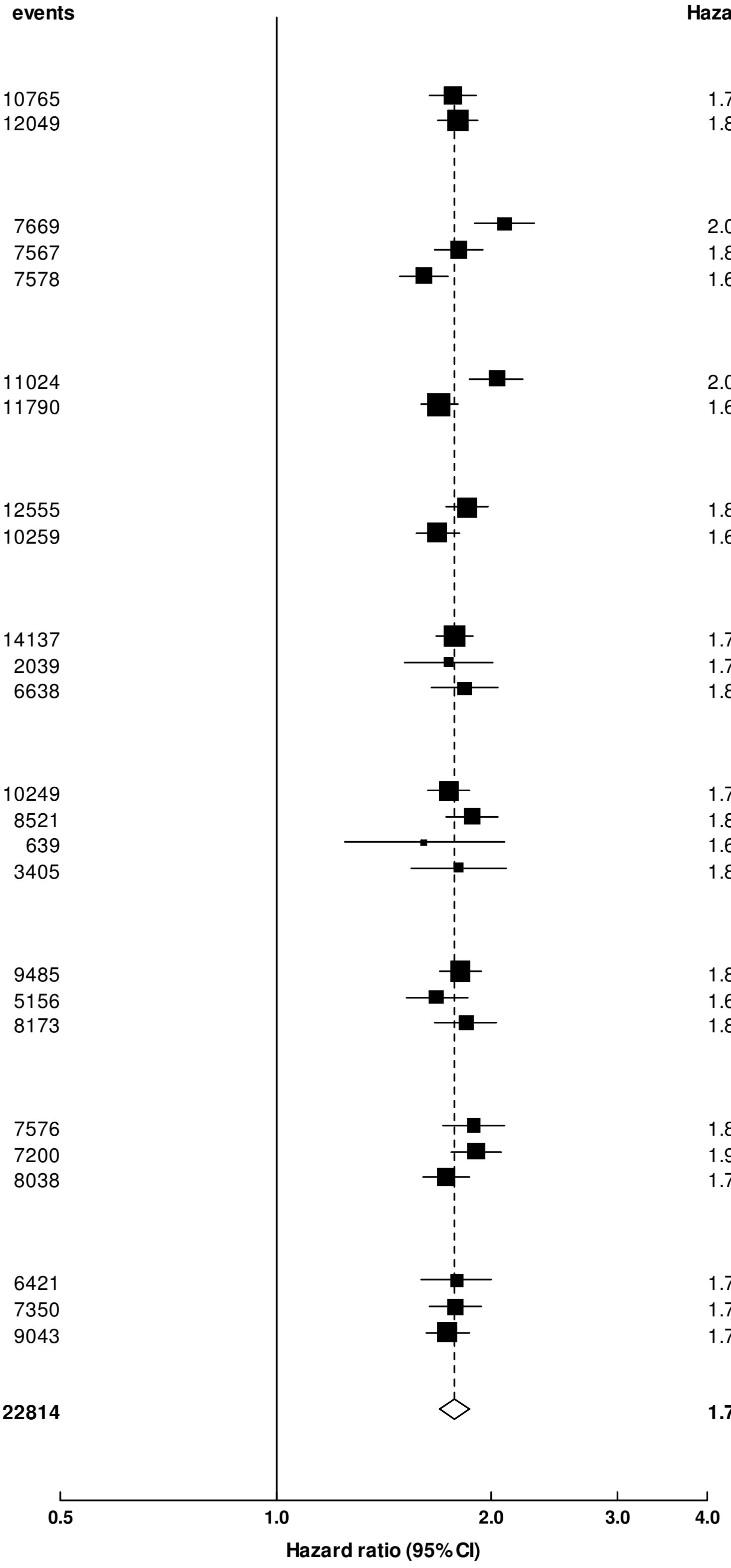

■ 95% CI

◇ 95% CI
