# Supplementary material for: Risks and Population Burden of Cardiovascular Diseases Associated with Diabetes in China: A Prospective Study of 0.5 Million Adults
Source: PLoS Med. 2016 Jul 5;13(7):e1002026. doi: 10.1371/journal.pmed.1002026 (PMC4933372; doi:10.1371/journal.pmed.1002026)
Supplement: S6 Fig — (PDF) [file pmed.1002026.s006.pdf]

S6 Fig. Adjusted hazard ratios for intracerebral haemorrhage by screen-detected diabetes status

Subgroup	No. of events	Hazard ratio (95% CI)
**Sex**		
Men	2375	1.38 (1.13 - 1.69)
Women	2045	1.00 (0.80 - 1.24)
Heterogeneity test p=0.03		
**Age (years)**		
35-59	1622	1.15 (0.89 - 1.51)
60-69	1392	1.16 (0.90 - 1.49)
70-79	1406	1.20 (0.94 - 1.54)
Trend test p=0.8		
**Study area**		
Rural	3510	1.26 (1.06 - 1.50)
Urban	910	0.98 (0.74 - 1.29)
Heterogeneity test p=0.1		
**Education (years)**		
<6	3189	1.18 (0.99 - 1.40)
6+	1231	1.13 (0.85 - 1.48)
Heterogeneity test p=0.8		
**Smoking**		
Never/occasional	2489	0.99 (0.81 - 1.22)
Ex-regular	386	1.10 (0.67 - 1.80)
Current regular	1519	1.42 (1.05 - 1.91)
Heterogeneity test p=0.2		
**Alcohol consumption**		
Never regular	2141	1.09 (0.88 - 1.35)
Occasional	1373	1.24 (0.95 - 1.62)
Ex-regular	169	1.16 (0.53 - 2.53)
Regular	737	1.24 (0.87 - 1.76)
Heterogeneity test p=0.9		
**Physical activity (MET hours/day)**		
<10.0	1542	1.23 (0.98 - 1.54)
10.0-14.9	887	1.10 (0.80 - 1.51)
15.0+	1991	1.13 (0.88 - 1.44)
Trend test p=0.6		
**Systolic blood pressure (mmHg)**		
<130	932	1.64 (1.12 - 2.39)
130-149	1258	1.34 (1.02 - 1.76)
150+	2230	1.07 (0.88 - 1.31)
Trend test p=0.04		
**BMI (kg/m2)**		
<22.0	1779	1.29 (0.97 - 1.72)
22.0-24.9	1328	1.27 (0.96 - 1.66)
25.0+	1313	1.10 (0.88 - 1.37)
Trend test p=0.3		
**Overall**	**4420**	**1.17 (1.01 - 1.36)**

Global heterogeneity test p=0.1

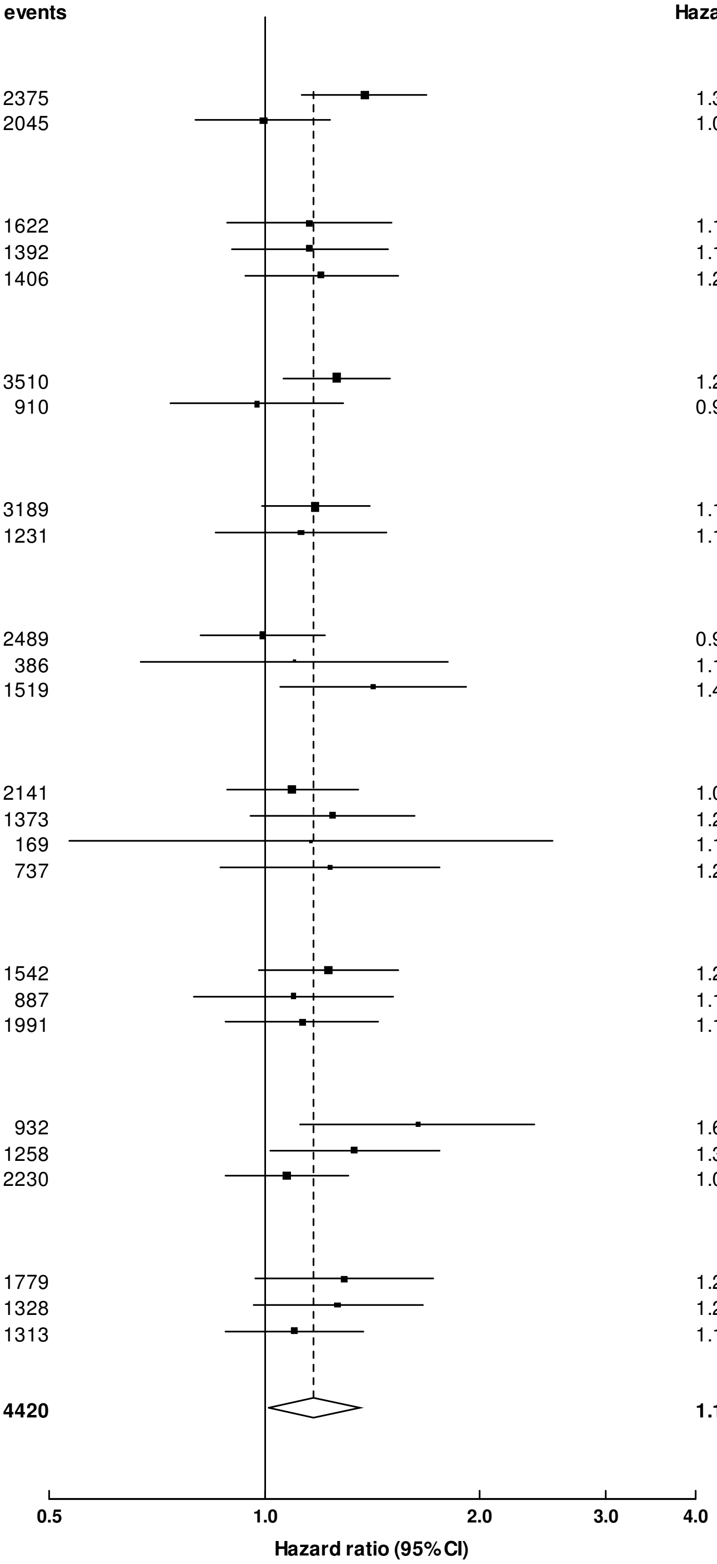

95% CI (square)

95% CI (diamond)
