# Supplementary material for: Risks and Population Burden of Cardiovascular Diseases Associated with Diabetes in China: A Prospective Study of 0.5 Million Adults
Source: PLoS Med. 2016 Jul 5;13(7):e1002026. doi: 10.1371/journal.pmed.1002026 (PMC4933372; doi:10.1371/journal.pmed.1002026)

S7 Fig. Adjusted hazard ratios for cardiovascular disease mortality by screen-detected diabetes status

Subgroup	No. of events	Hazard ratio (95% CI)
**Sex**		
Men	3873	1.77 (1.55 - 2.03)
Women	2909	1.56 (1.36 - 1.80)
Heterogeneity test p=0.2		
**Age (years)**		
35-59	1505	1.65 (1.31 - 2.07)
60-69	1996	1.69 (1.42 - 2.02)
70-79	3281	1.64 (1.43 - 1.88)
Trend test p=0.9		
**Study area**		
Rural	5053	1.62 (1.43 - 1.84)
Urban	1729	1.75 (1.50 - 2.05)
Heterogeneity test p=0.4		
**Education (years)**		
<6	5134	1.60 (1.43 - 1.80)
6+	1648	1.80 (1.50 - 2.16)
Heterogeneity test p=0.3		
**Smoking**		
Never/occasional	3510	1.62 (1.42 - 1.85)
Ex-regular	734	1.57 (1.16 - 2.11)
Current regular	2538	1.79 (1.51 - 2.13)
Heterogeneity test p=0.6		
**Alcohol consumption**		
Never regular	3282	1.61 (1.41 - 1.86)
Occasional	2106	1.71 (1.43 - 2.05)
Ex-regular	331	1.89 (1.20 - 2.96)
Regular	1063	1.59 (1.23 - 2.06)
Heterogeneity test p=0.9		
**Physical activity (MET hours/day)**		
<10.0	3113	1.63 (1.42 - 1.87)
10.0-14.9	1257	1.79 (1.45 - 2.23)
15.0+	2412	1.62 (1.35 - 1.95)
Trend test p=0.9		
**Systolic blood pressure (mmHg)**		
<130	1594	1.93 (1.51 - 2.47)
130-149	1926	2.21 (1.86 - 2.62)
150+	3262	1.43 (1.25 - 1.64)
Trend test p=0.002		
**BMI (kg/m2)**		
<22.0	2909	1.74 (1.45 - 2.10)
22.0-24.9	1945	1.65 (1.37 - 2.00)
25.0+	1928	1.72 (1.48 - 1.99)
Trend test p=0.9		
**Overall**	**6782**	**1.66 (1.51 - 1.83)**

Global heterogeneity test p=0.3

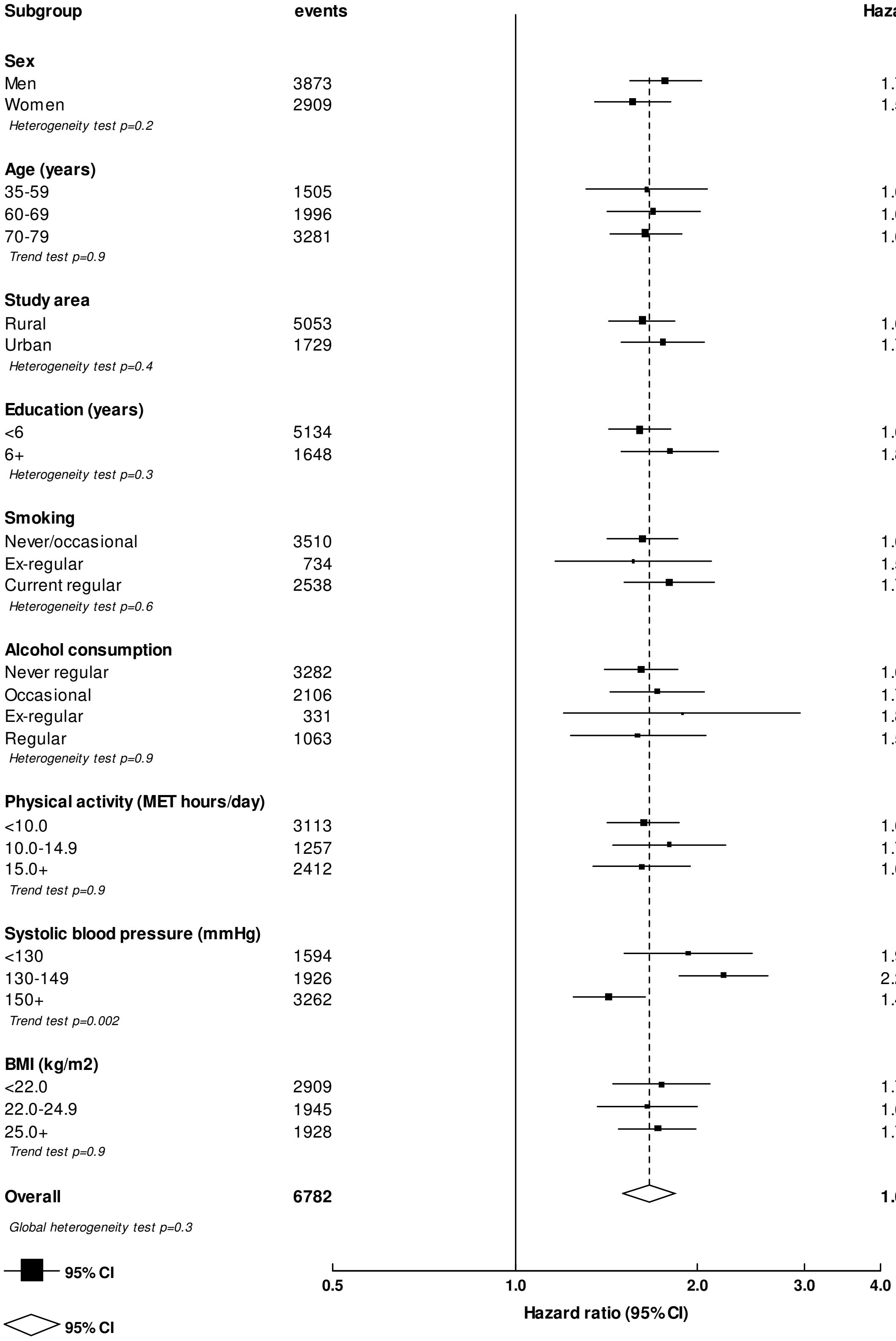

Supplement: S7 Fig — (PDF) [file pmed.1002026.s007.pdf]
