# Supplementary material for: Risks and Population Burden of Cardiovascular Diseases Associated with Diabetes in China: A Prospective Study of 0.5 Million Adults
Source: PLoS Med. 2016 Jul 5;13(7):e1002026. doi: 10.1371/journal.pmed.1002026 (PMC4933372; doi:10.1371/journal.pmed.1002026)

S8 Fig. Adjusted hazard ratios for major occlusive vascular disease by screen-detected diabetes status

Subgroup	No. of events	Hazard ratio (95% CI)
**Sex**		
Men	10589	1.54 (1.41 - 1.67)
Women	11643	1.47 (1.37 - 1.59)
Heterogeneity test p=0.4		
**Age (years)**		
35-59	7626	1.73 (1.56 - 1.91)
60-69	7289	1.48 (1.35 - 1.63)
70-79	7317	1.35 (1.23 - 1.48)
Trend test p<0.001		
**Study area**		
Rural	10950	1.69 (1.54 - 1.84)
Urban	11282	1.42 (1.32 - 1.52)
Heterogeneity test p=0.003		
**Education (years)**		
<6	12279	1.48 (1.37 - 1.60)
6+	9953	1.51 (1.39 - 1.64)
Heterogeneity test p=0.8		
**Smoking**		
Never/occasional	13717	1.52 (1.42 - 1.62)
Ex-regular	1926	1.38 (1.15 - 1.66)
Current regular	6589	1.52 (1.36 - 1.70)
Heterogeneity test p=0.6		
**Alcohol consumption**		
Never regular	9814	1.45 (1.33 - 1.57)
Occasional	8352	1.49 (1.36 - 1.64)
Ex-regular	597	1.47 (1.02 - 2.13)
Regular	3469	1.65 (1.44 - 1.88)
Heterogeneity test p=0.4		
**Physical activity (MET hours/day)**		
<10.0	9072	1.40 (1.29 - 1.52)
10.0-14.9	5026	1.52 (1.36 - 1.71)
15.0+	8134	1.67 (1.51 - 1.85)
Trend test p=0.006		
**Systolic blood pressure (mmHg)**		
<130	7416	1.56 (1.38 - 1.77)
130-149	6984	1.66 (1.51 - 1.83)
150+	7832	1.44 (1.33 - 1.57)
Trend test p=0.1		
**BMI (kg/m2)**		
<22.0	6303	1.58 (1.38 - 1.81)
22.0-24.9	7100	1.47 (1.33 - 1.64)
25.0+	8829	1.44 (1.34 - 1.56)
Trend test p=0.3		
**Overall**	**22232**	**1.50 (1.41 - 1.58)**

Global heterogeneity test p<0.001

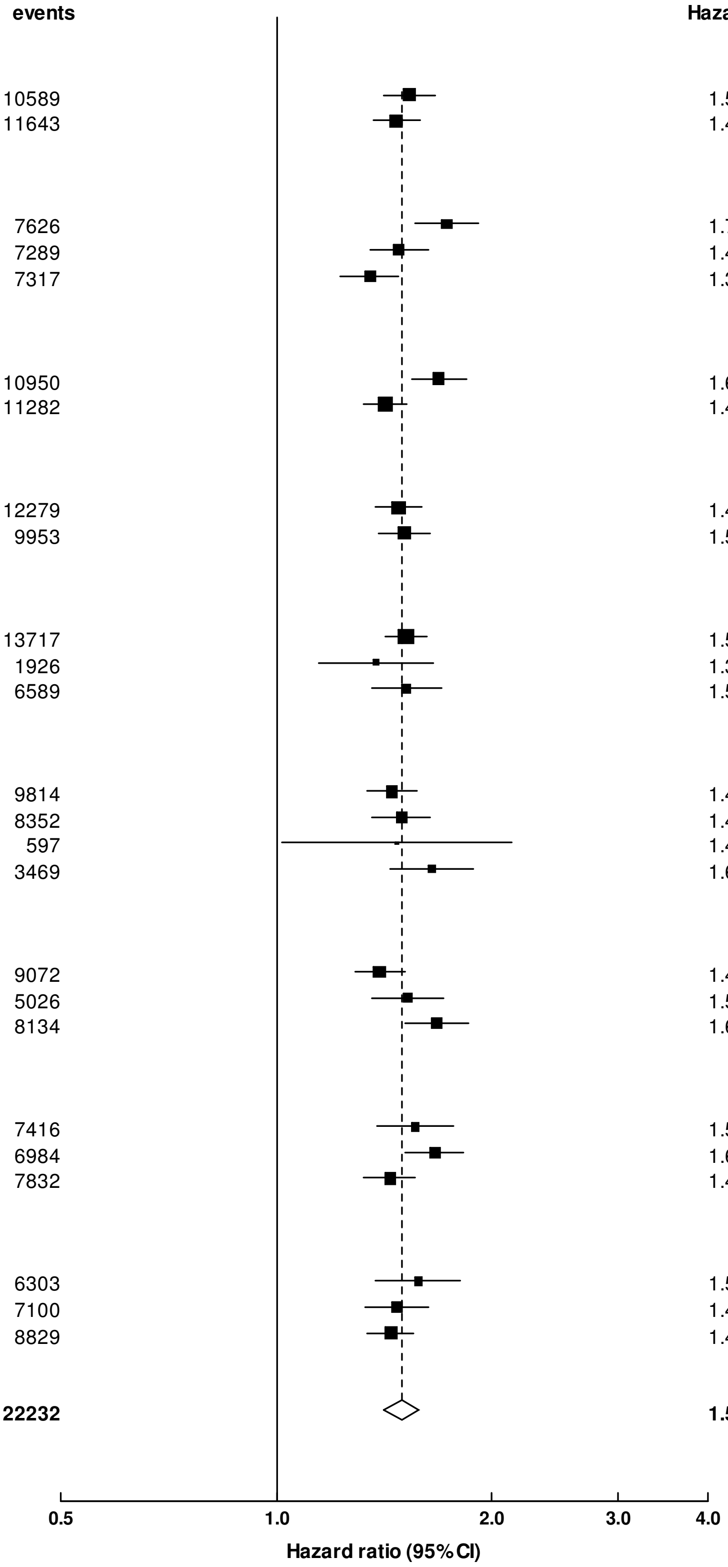

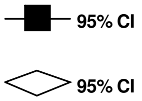

Supplement: S8 Fig — (PDF) [file pmed.1002026.s008.pdf]
