# Supplementary material for: Risks and Population Burden of Cardiovascular Diseases Associated with Diabetes in China: A Prospective Study of 0.5 Million Adults
Source: PLoS Med. 2016 Jul 5;13(7):e1002026. doi: 10.1371/journal.pmed.1002026 (PMC4933372; doi:10.1371/journal.pmed.1002026)
Supplement: S1 Table — (PDF) [file pmed.1002026.s009.pdf]

S1 Table. Adjusted hazard ratios for incident cardiovascular diseases by diabetes status, additionally adjusted for adiposity

Outcomes	Diabetes		No diabetes			
	Events	Rate per 100 000 person years[a]	Events	Rate per 100 000 person years[a]	HR[b]	95%CI
**Self-reported diabetes[c]**						
**Cardiovascular disease mortality**	571	351.1	6345	133.5	2.07	1.89-2.26
**Major occlusive vascular disease**	1938	1177.8	20876	536.8	1.71	1.63-1.79
**Ischaemic heart disease**						
Fatal MI	127	88.1	1237	25.7	2.58	2.13-3.12
Non-fatal MI	122	78.2	1087	27.3	2.10	1.73-2.56
Major coronary event	368	211.9	3097	69.2	2.37	2.12-2.65
**Stroke**						
Ischaemic stroke						
Fatal	43	24.5	416	8.6	2.21	1.59-3.07
Non-fatal	1589	952.6	17730	463.6	1.60	1.52-1.69
Any	1632	977.2	18146	472.1	1.62	1.53-1.70
Intracerebral haemorrhage						
Fatal	100	109.6	1891	42.6	1.62	1.32-2.00
Non-fatal	92	66.3	2340	61.8	1.00	0.81-1.23
Any	192	175.9	4231	104.4	1.25	1.08-1.45
Total stroke						
Fatal	168	154.8	2575	56.9	1.81	1.54-2.13
Non-fatal	1749	1068.6	21157	553.5	1.54	1.47-1.62
Any	1917	1223.3	23732	610.4	1.56	1.49-1.64
**Screen-detected diabetes[c]**						
**Cardiovascular disease mortality**	437	294.0	6345	133.5	1.66	1.51-1.83
**Major occlusive vascular disease**	1356	1021.1	20876	536.8	1.44	1.36-1.52
**Ischaemic heart disease**						
Fatal MI	81	60.6	1237	25.7	1.61	1.28-2.03
Non-fatal MI	68	52.3	1087	27.3	1.44	1.13-1.85
Major coronary event	213	140.0	3097	69.2	1.57	1.36-1.81
**Stroke**						
Ischaemic stroke						
Fatal	33	19.3	416	8.6	1.84	1.28-2.64
Non-fatal	1144	869.5	17730	463.6	1.41	1.33-1.50
Any	1177	888.8	18146	472.1	1.42	1.34-1.51
Intracerebral haemorrhage						
Fatal	116	96.8	1891	42.6	1.65	1.36-2.00
Non-fatal	73	69.1	2340	61.8	0.81	0.64-1.03
Any	189	165.9	4231	104.4	1.18	1.02-1.37
Total stroke						
Fatal	168	129.1	2575	56.9	1.69	1.44-1.98
Non-fatal	1268	981.7	21157	553.5	1.34	1.27-1.42
Any	1436	1110.8	23732	610.4	1.37	1.30-1.45

[a]Age-, sex- and study area-standardised rates; [b]Stratified by age, sex and study area and adjusted for education, smoking, alcohol, physical activity, systolic blood pressure and waist-to-hip ratio; [c]Reference group is individuals without self-reported or screen-detected diabetes.
Events classified as fatal if death from any cause recorded within 28 days of disease onset.
CI, confidence interval; HR, hazard ratio; MI, myocardial infarction.
