# Supplementary material for: Risks and Population Burden of Cardiovascular Diseases Associated with Diabetes in China: A Prospective Study of 0.5 Million Adults
Source: PLoS Med. 2016 Jul 5;13(7):e1002026. doi: 10.1371/journal.pmed.1002026 (PMC4933372; doi:10.1371/journal.pmed.1002026)
Supplement: S2 Table — (PDF) [file pmed.1002026.s010.pdf]

S2 Table. Adjusted hazard ratios for incident cardiovascular diseases by self-reported diabetes status excluding participants who developed incident diabetes during follow-up (n= 8896)

**Outcomes**	**HR[a]**	**95% CI**
**Self-reported diabetes[b]**		
**Cardiovascular disease mortality**	2.05	1.88-2.24
**Major occlusive vascular disease**	1.82	1.74-1.91
**Ischaemic heart disease**		
Fatal MI	2.63	2.18-3.18
Non-fatal MI	2.31	1.90-2.80
Major coronary event	2.47	2.20-2.76
**Stroke**		
Ischaemic stroke		
Fatal	2.19	1.58-3.04
Non-fatal	1.72	1.63-1.82
Any	1.73	1.64-1.82
Intracerebral haemorrhage		
Fatal	1.58	1.29-1.94
Non-fatal	1.00	0.81-1.24
Any	1.24	1.07-1.44
Total stroke		
Fatal	1.76	1.50-2.07
Non-fatal	1.64	1.56-1.73
Any	1.65	1.57-1.73

[a]Stratified by age, sex and study area and adjusted for education, smoking, alcohol, physical activity and systolic blood pressure; [b]Reference group is individuals without self-reported or screen-detected diabetes.
Events classified as fatal if death from any cause within 28 days.
CI, confidence interval; HR, hazard ratio; MI, myocardial infarction.
