# Supplementary material for: Risks and Population Burden of Cardiovascular Diseases Associated with Diabetes in China: A Prospective Study of 0.5 Million Adults
Source: PLoS Med. 2016 Jul 5;13(7):e1002026. doi: 10.1371/journal.pmed.1002026 (PMC4933372; doi:10.1371/journal.pmed.1002026)
Supplement: S3 Table — (PDF) [file pmed.1002026.s011.pdf]

S3 Table. Adjusted hazard ratios for incident cardiovascular diseases by diabetes status

Outcomes	Diabetes		No diabetes		Model A[b]		Model B[c]	
	Events	Rate per 100 000 person years[a]	Events	Rate per 100 000 person years[a]	HR	95%CI	HR	95%CI
**Self-reported diabetes[d]**								
Other ischaemic heart disease[e]	1244	872.6	18499	491.2	1.52	1.43-1.61	1.39	1.31-1.48
Other cerebrovascular disease[f]	350	284.6	7884	214.0	1.28	1.15-1.42	1.23	1.10-1.37
**Screen-detected diabetes[d]**								
Other ischaemic heart disease[e]	874	625.6	18499	491.2	1.25	1.17-1.34	1.17	1.09-1.25
Other cerebrovascular disease[f]	298	274.8	7884	214.0	1.26	1.12-1.41	1.22	1.08-1.37

[a]Age-, sex- and study area-standardised rates; [b]Model A: stratified by age, sex and study area; [c]Model B: additionally adjusted for education, smoking, alcohol, physical activity and systolic blood pressure; [d]Reference group is individuals without self-reported or screen-detected diabetes; [e]All ischaemic heart disease (ICD-10 I20-25) excluding ischaemic heart disease death or non-fatal myocardial infarction; [f]All cerebrovascular disease (ICD-10 I60-69) excluding total stroke.
CI, confidence interval; HR, hazard ratio.
