# Supplementary material for: Risks and Population Burden of Cardiovascular Diseases Associated with Diabetes in China: A Prospective Study of 0.5 Million Adults
Source: PLoS Med. 2016 Jul 5;13(7):e1002026. doi: 10.1371/journal.pmed.1002026 (PMC4933372; doi:10.1371/journal.pmed.1002026)
Supplement: S1 Text — (DOCX) [file pmed.1002026.s012.docx]

**S1 Text. Original analysis plan and modifications for the presented analyses**

**Objectives**

1. Understand the association between prevalent, self-reported diabetes and other cardiovascular disease (CVD) risk factors
2. Understand the association between screen-detected diabetes and other CVD risk factors
3. Characterise the association of self-reported diabetes with risk of incident CVDs, including ischaemic heart and stroke subtypes
4. Examine how associations of self-reported diabetes with incident CVDs are modified by other factors e.g. age, sex
5. Characterise the association of screen-detected diabetes with risk of incident CVDs, including ischaemic heart disease (IHD) and stroke subtypes
6. Examine how associations of screen-detected diabetes with incident CVDs are modified by other factors e.g. age, sex
7. Examine how associations of diabetes with incident CVDs differ by presence of other CVD risk factors
8. Estimate number of CVD deaths attributable to diabetes in adult Chinese population

**Exclusions**

- Missing body mass index (BMI) data (n=2)
- Missing, implausible or extreme systolic blood pressure, diastolic blood pressure, height, waist circumference, hip circumference, waist:hip, BMI (n=1079)
- Prevalent IHD and stroke/transient ischaemic attack (n=23 050)

Analyses thus based on a remaining population of 488 760

**Exposures**

- Self-reported diabetes
  - Comparator group is no self-reported diabetes which will contain some individuals with screen-detected diabetes
- Screen-detected diabetes
  - Defined as no self-reported history of diabetes with a blood glucose level ≥7.0mmol/L and a fasting time ≥8 hours, a blood glucose level ≥11.1mmol/L and a fasting time <8 hours, or a fasting blood glucose level ≥7.0mmol/L
  - Comparator group is no screen-detected or self-reported diabetes

**Outcomes**

- IHD
- Major coronary event
- Myocardial infarction- fatal, non-fatal, fatal and non-fatal
- Other IHD
- Cerebrovascular disease
- Total stroke- fatal, non-fatal, fatal and non-fatal
- Ischaemic stroke- fatal, non-fatal, fatal and non-fatal
- Intracerebral haemorrhage- fatal, non-fatal, fatal and non-fatal
- Other cerebrovascular disease
- Major occlusive vascular disease
- CVD mortality

**Subgroup analyses**

- Participants reporting taking and not taking diabetic medications (insulin or oral hypoglycaemic medications)
- Participants reporting taking and not taking aspirin (if sufficient numbers)
- Participants reporting taking and not taking statin (if sufficient numbers)
- Type 1 and type 2 diabetes separately (if sufficient numbers) using the following algorithm to differentiate:
  - Define type 2 diabetes as disease onset at ≥35y or no self-reported use of insulin
  - Otherwise classify as type 1 diabetes

**Additional analyses**

- Exclude the first four years of follow-up
- Exclude incident diabetes cases
- Stratify by duration of diabetes to examine its effect on associations between self-reported diabetes and incident CVDs
- Examine risk within participants with diabetes according to number of other CVD risk factors present (smoking, adiposity, physical inactivity, hypertension)
- Population attributable risk to estimate number of CVD deaths attributable to diabetes based on Global Burden of Disease mortality data and diabetes prevalence from 2010 survey (Xu et al, JAMA)

**Adjustment for confounding**

Based on a priori knowledge of potential confounding factors and their associations with diabetes and with incident stroke and IHD:

- Adjusted for age, sex (where appropriate) and study area
- Model A: age, sex (where appropriate), study area, education, smoking, alcohol
- Model B: additional adjustment for physical activity and systolic blood pressure
- Model C: additional adjustment for waist:hip

**Statistical model**

The Cox proportional hazards model will be used (after initial investigation to ensure assumptions are met).

**Modifications to the original analysis plan for the presented analyses**

We did not publish or pre-register a protocol for these analyses. However, the presented analyses are consistent with those that were planned in the original analysis plan described above, with the exception of the modifications presented below.

| **Original analysis plan** | **Presented analyses** | **Justification** |
| --- | --- | --- |
| Comparator group for self-reported diabetes analyses: individuals without self-reported diabetes at baseline | Comparator group for self-reported diabetes analyses: individuals without self-reported or screen-detected diabetes at baseline | Exclusion of individuals with both self-reported and screen-detected diabetes from the comparator group for the self-reported diabetes analyses meant that the comparator groups for self-reported and screen-detected diabetes analyses were the same. This enabled more appropriate comparisons of cardiovascular risks associated with self-reported and with screen-detected diabetes. In addition, it enabled more appropriate comparisons of the risks of cardiovascular diseases in individuals with and without diabetes. |
