# Supplementary material for: Risks and Population Burden of Cardiovascular Diseases Associated with Diabetes in China: A Prospective Study of 0.5 Million Adults
Source: PLoS Med. 2016 Jul 5;13(7):e1002026. doi: 10.1371/journal.pmed.1002026 (PMC4933372; doi:10.1371/journal.pmed.1002026)
Supplement: S2 Text — (DOC) [file pmed.1002026.s013.doc]

STROBE Statement—Checklist of items that should be included in reports of ***cohort studies***

|  | Item No | Recommendation |
| --- | --- | --- |
| **Title and abstract** | 1 | (*a*) Indicate the study’s design with a commonly used term in the title or the abstract  ***Done.***  ***The prospective design is described in the title: “Emerging burden of diabetes-related cardiovascular disease in China: a 7-year prospective study of 0.5 million adults”. It is also described in the “Methods and findings section” of the Abstract: “A nationwide prospective study recruited 512 891 men and women aged 30-79 years in 2004-8 from 10 diverse localities across China”.*** |
|  |  | (*b*) Provide in the abstract an informative and balanced summary of what was done and what was found  ***Done.***  ***A summary of what was done and what was found is provided in the “Methods and findings section” of the Abstract: “A nationwide prospective study recruited 512 891 men and women aged 30-79 years in 2004-8 from 10 diverse localities across China. During ~7 years of follow-up, 7353 cardiovascular deaths and 25 451 non-fatal major cardiovascular events were recorded among 488 760 participants without prior cardiovascular diseases at baseline. Cox regression yielded adjusted hazard ratios (HRs) comparing disease risks in individuals with diabetes to those without. Overall, 5.4% (n=26 335) of participants had self-reported (2.7%) or screen-detected (2.7%) diabetes. Individuals with self-reported diabetes had an adjusted HR of 2.07 (95% CI 1.90-2.26) for cardiovascular mortality. There were significant excess risks of major coronary event (MCE: 2.44, 2.18-2.73), ischaemic stroke (IS: 1.68, 1.60-1.77) and intracerebral haemorrhage (ICH: 1.24, 1.07-1.44). Screen-detected diabetes was also associated with significant, though more modest, excess cardiovascular risks, with corresponding HRs of 1.66 (1.51-1.83), 1.62 (1.40-1.86), 1.48 (1.40-1.57) and 1.17 (1.01-1.36), respectively. Among individuals with diabetes, cardiovascular risk increased progressively with duration of diabetes and number of other presenting modifiable cardiovascular risk factors. Assuming a causal association, diabetes now accounts for ~0.5 million (489 676 95% CI 335 777-681 202) cardiovascular deaths annually in China.”*** |
| Introduction | | |
| Background/rationale | 2 | Explain the scientific background and rationale for the investigation being reported  ***Done.***  ***The scientific background and rationale for the study are described in the first two paragraphs of the Introduction: “Worldwide about 400 million people have diabetes, and the prevalence is increasing rapidly in both developed and developing countries. Previous studies of mostly Western populations have shown that diabetes is typically associated with a two-fold increased risk of ischaemic heart disease (IHD). Uncertainty remains, however, about whether similar excess risk applies to other populations, and about the strength of the associations of diabetes with stroke, and particularly stroke subtypes. Appropriate understanding of these issues is of considerable relevance to China, where stroke rates are high.***  ***Since the 1980s there has been a rapid and substantial increase in the prevalence of diabetes in China, which now affects ~10% of adults. Compared with those in the West, individuals with diabetes in China have tended to be leaner and to have worse pancreatic beta-cell function, which may result in greater susceptibility to microvascular complications and cancer than to macrovascular complications. Despite the growing diabetes epidemic, there is limited evidence about the association of diabetes with cardiovascular disease in China, with previous studies limited by potentially out-dated risk estimates, highly selected study populations or relatively small size, and lack of proper investigation of the relevance of diabetes duration and other modifiable factors, such as smoking, adiposity, blood pressure and physical activities, which frequently differ between China and the West.”*** |
| Objectives | 3 | State specific objectives, including any prespecified hypotheses  ***Done.***  ***The objectives of the study are described in the third paragraph of the Introduction: “The aim of the present study is to examine the associations of diabetes, both self-reported doctor-diagnosed and screen-detected, with risks of major cardiovascular diseases, including IHD and stroke subtypes.”*** |
| Methods | | |
| Study design | 4 | Present key elements of study design early in the paper  ***Done.***  ***All key elements of the study design are presented in the third paragraph of the Introduction: “We report relevant findings from a large prospective study (the China Kadoorie Biobank, CKB) of 0.5 million adults, established during 2004-08 across 10 diverse areas of China”.***  ***They are also presented in paragraphs one and two of the Methods: “All participants provided written consent prior to participation, including permission for follow-up. Ethics approval was obtained from Oxford University, the China National Centre for Disease Control and Prevention (CDC) and the 10 study areas’ local CDCs.”; “Details of the CKB design, methods and population have been reported previously. Briefly, the 2004-8 baseline survey took place in 10 localities across China (five urban and five rural, S1 Fig), chosen to provide diversity in exposure and disease patterns, additionally taking account of logistical considerations, including population stability, death and disease registry quality, and local capacity. All residents aged 35-74 years from 100-150 administrative units (rural villages or urban residential committees) in each area were invited to attend survey clinics. Overall, ~30% responded, and 512 891 individuals were enrolled (including a few slightly outside the 35-74 years range).”*** |
| Setting | 5 | *Describe the setting, locations, and relevant dates, including periods of recruitment, exposure, follow-up, and data collection*  ***Done.***  ***The setting, locations, and relevant dates, including periods of recruitment, exposure, follow-up, and data collection are reported in “Study population”, “Data collection” and “Follow-up for mortality and morbidity” sections of the methods, as below:***  ***“…..the 2004-8 baseline survey took place in 10 localities across China (five urban and five rural, S1 Fig), chosen to provide diversity in exposure and disease patterns, additionally taking account of logistical considerations, including population stability, death and disease registry quality, and local capacity.” Methods, paragraph two.***  ***“Data collection***  ***At study assessment clinics, trained health workers administered laptop-based questionnaires which collected data on socio-demographic status, tobacco, alcohol consumption, diet, physical activity (related to leisure, household work, occupation and commuting), medical history and, among participants reporting a history of diabetes, IHD, stroke/transient ischaemic attack (TIA) or hypertension, current use of medication; measured height, weight, waist and hip circumference, and blood pressure; and took non-fasting venous blood samples (with the time since last food recorded) for storage and immediate on-site testing for plasma glucose levels using the SureStep Plus meter (LifeScan, Milipitas, CA, USA). Participants without self-reported diabetes with a plasma glucose level of 7.8-11.0 mmol/L were invited to undergo fasting glucose testing (using the same measurement technique) the following day. Every 4-5 years a 5-6% random sample of surviving participants was resurveyed, collating the same data as at baseline with certain additions.***  ***Assessment of diabetes status***  ***Participants answering “Yes” to the question, “Has a doctor ever told you that you had had diabetes?” at baseline were defined as having self-reported diabetes; among them, information about age at diagnosis and current medication use was collected. Screen-detected diabetes was defined as not having self-reported diabetes, but having a random plasma glucose (RPG) level ≥7.0 mmol/L if time since last food ≥8 hours, or ≥11.1 mmol/L if time since last food <8 hours, or a fasting plasma glucose level ≥7.0 mmol/L on subsequent testing. Participants with screen-detected diabetes were provided with a referral letter and advised to seek formal medical consultation.***  ***Follow-up for mortality and morbidity***  ***Vital status of each participant was obtained periodically from China CDC’s Disease Surveillance Points (DSP), checked annually against local residential and health insurance records, and by active confirmation through street committee or village administrators. Causes of death from official death certificates were ICD-10 coded by trained DSP staff, blinded to baseline information. Information on non-fatal outcomes was collected through linkage with established disease surveillance systems (for cancer, IHD, stroke and diabetes) and, via unique national ID, with the national health insurance system, which records details of ICD-10 coded hospitalisations in all study areas.” Methods, paragraphs three, four and five.*** |
| Participants | 6 | (*a*) Give the eligibility criteria, and the sources and methods of selection of participants. Describe methods of follow-up  ***Done.***  ***The eligibility criteria, and the sources and methods of selection of participants are presented in the “Study population” section of the Methods: “All residents aged 35-74 years from 100-150 administrative units (rural villages or urban residential committees) in each area were invited to attend survey clinics. Overall, ~30% responded, and 512 891 individuals were enrolled (including a few slightly outside the 35-74 years range).” Methods, paragraph two.***  ***Methods of follow-up are described in the “Follow-up for mortality and morbidity” section of the Methods: “Vital status of each participant was obtained periodically from China CDC’s Disease Surveillance Points (DSP), checked annually against local residential and health insurance records, and by active confirmation through street committee or village administrators. Causes of death from official death certificates were ICD-10 coded by trained DSP staff, blinded to baseline information. Information on non-fatal outcomes was collected through linkage with established disease surveillance systems (for cancer, IHD, stroke and diabetes) and, via unique national ID, with the national health insurance system, which records details of ICD-10 coded hospitalisations in all study areas.” Methods, paragraph five.*** |
|  |  | (*b*) For matched studies, give matching criteria and number of exposed and unexposed  ***N/A*** |
| Variables | 7 | Clearly define all outcomes, exposures, predictors, potential confounders, and effect modifiers. Give diagnostic criteria, if applicable  ***Done.***  ***Exposures, including diagnostic criteria for diabetes, are defined in the “Assessment of diabetes status” section of the Methods: “Participants answering “Yes” to the question, “Has a doctor ever told you that you had had diabetes?” at baseline were defined as having self-reported diabetes; among them, information about age at diagnosis and current medication use was collected. Screen-detected diabetes was defined as not having self-reported diabetes, but having a random plasma glucose (RPG) level ≥7.0 mmol/L if time since last food ≥8 hours, or ≥11.1 mmol/L if time since last food <8 hours, or a fasting plasma glucose level ≥7.0 mmol/L on subsequent testing.*** ***Participants with screen-detected diabetes were provided with a referral letter and advised to seek formal medical consultation.” Methods, paragraph four.***  ***Outcomes are defined in the “Follow-up for mortality and morbidity” section of the Methods: “For the present study, the primary endpoints were cardiovascular death (ICD-10 I00-25, I27-88, I95-99), myocardial infarction (MI, I21-23), major coronary event (MCE: non-fatal MI or fatal IHD [I20-25]), ischaemic stroke (IS, I63), intracerebral haemorrhage (ICH, I61), total stroke (TS, I60, I61, I63, I64), and major occlusive vascular disease (MOVD: IS or MCE).” Methods, paragraph six.***  ***Potential confounders and effect modifiers are defined in the “Statistical analysis” section of the Methods: “Cox regression yielded hazard ratios (HR) for diabetes versus not, stratified by age-at-risk (5-year age groups), study area and, where appropriate, sex, and adjusted for education (no formal education, primary school, middle school, high school, college/university), smoking (never, occasional, ex-regular, current regular), alcohol (never, occasional intake, ex-regular, reduced intake, weekly intake), systolic blood pressure (SBP) (9 groups), and physical activity (5 groups). Sensitivity analyses were performed adjusting for age, SBP and physical activity as continuous variables” and “Adjusted HRs were calculated across strata of other cardiovascular risk factors and duration of diabetes at baseline….. In separate analyses, risk estimates were also adjusted for adiposity.” Methods, paragraphs eight and nine.*** |
| Data sources/ measurement | 8* | For each variable of interest, give sources of data and details of methods of assessment (measurement). Describe comparability of assessment methods if there is more than one group  ***Done.***  ***Sources of data and details of methods of assessment of baseline and resurvey variables are described in the “Data collection” section of the Methods: “At study assessment clinics, trained health workers administered laptop-based questionnaires which collected data on socio-demographic status, tobacco, alcohol consumption, diet, physical activity (related to leisure, household work, occupation and commuting), medical history and, among participants reporting a history of diabetes, IHD, stroke/transient ischaemic attack (TIA) or hypertension, current use of medications; measured height, weight, waist and hip circumference, and blood pressure; and took non-fasting venous blood samples (with the time since last food recorded) for storage and immediate on-site testing for plasma glucose levels using the SureStep Plus meter (LifeScan, Milipitas, CA, USA). Participants without self-reported diabetes with a plasma glucose level of 7.8-11.0 mmol/L were invited to undergo fasting glucose testing (using the same measurement technique) the following day. Every 4-5 years a 5-6% random sample of surviving participants was resurveyed, collating the same data as at baseline with certain additions.” Methods, paragraph three.***  ***Sources of data and details of methods of assessment of outcome data re described in the “Follow-up for morbidity and mortality” section of the Methods: “Vital status of each participant was obtained periodically from China CDC’s Disease Surveillance Points (DSP), checked annually against local residential and health insurance records, and by active confirmation through street committee or village administrators. Causes of death from official death certificates were ICD-10 coded by trained DSP staff, blinded to baseline information. Information on non-fatal outcomes was collected through linkage with established disease surveillance systems (for cancer, IHD, stroke and diabetes) and, via unique national ID, with the national health insurance system, which records details of ICD-10 coded hospitalisations in all study areas.” Methods, paragraph five.*** |
| Bias | 9 | Describe any efforts to address potential sources of bias  ***As described in the Methods, recruitment was approximately random, limiting selection bias (paragraph two): “All residents aged 35-74 years from 100-150 administrative units (rural villages or urban residential committees) in each area were invited to attend survey clinics.” Participants with self-reported diabetes were examined separately in an attempt to avoid potential biases resulting from a known diagnosis of diabetes, including biases from lifestyle changes and treatment following diagnosis (Methods, paragraph eight): “Analyses were done separately for self-reported and screen-detected diabetes with a common reference group consisting of individuals without self-reported or screen-detected diabetes.” Outcomes were derived from multiple different data sources, limiting biases inherent in the use of a single data source, and coding of disease events was blinded, further reducing the risk of bias (Methods, paragraph five): “Vital status of each participant was obtained periodically from China CDC’s Disease Surveillance Points (DSP), checked annually against local residential and health insurance records, and by active confirmation through street committee or village administrators. Causes of death from official death certificates were ICD-10 coded by trained DSP staff, blinded to baseline information. Information on non-fatal outcomes was collected through linkage with established disease surveillance systems (for cancer, IHD, stroke and diabetes) and, via unique national ID, with the national health insurance system, which records details of ICD-10 coded hospitalisations in all study areas.”*** |
| Study size | 10 | Explain how the study size was arrived at  ***Done.***  ***Analyses were based on the entire CKB population excluding participants as described in the “Statistical analysis” sections of the Methods: “The main analyses excluded participants reporting prior doctor-diagnosed IHD (n=15 472, 3.0%) or stroke/TIA (n=8884, 1.7%) at baseline. A further 1081 (0.2%) participants with missing, implausible or extreme values for blood pressure, height, waist circumference, hip circumference, waist-to-hip ratio or body mass index (BMI) were excluded, leaving 488 760 (199 896 men, 288 864 women) for the present analyses”. Methods, paragraph seven.*** |
| Quantitative variables | 11 | Explain how quantitative variables were handled in the analyses. If applicable, describe which groupings were chosen and why  ***Done.***  ***The approach to handling quantitative variables is described in “Assessment of diabetes status” section of the Methods (paragraph four): “Screen-detected diabetes was defined as not having self-reported diabetes, but having a random plasma glucose (RPG) level ≥7.0mmol/L if time since last food ≥8 hours, or ≥11.1mmol/L if time since last food <8 hours, or a fasting plasma glucose level ≥7.0mmol/L on subsequent testing”. It is also described in the “Statistical analyses” section of the Methods (paragraph eight): “Cox regression yielded hazard ratios (HR) for diabetes versus not, stratified by age-at-risk (5-year age groups)…..systolic blood pressure (SBP) (9 groups), and physical activity (5 groups). Sensitivity analyses were performed adjusting for age, SBP and physical activity as continuous variables”.*** |
| Statistical methods | 12 | (*a*) Describe all statistical methods, including those used to control for confounding  ***Done.***  ***The statistical methods used are described throughout the “Statistical analyses” section of the Methods: “The main analyses excluded participants reporting prior doctor-diagnosed IHD (n=15 472, 3.0%) or stroke/TIA (n=8884, 1.7%) at baseline. A further 1081 (0.2%) participants with missing, implausible or extreme values for blood pressure, height, waist circumference, hip circumference, waist-to-hip ratio or body mass index (BMI) were excluded, leaving 488 760 (199 896 men, 288 864 women) for the present analyses.***  ***Analyses were done separately for self-reported and screen-detected diabetes with a common reference group consisting of individuals without self-reported or screen-detected diabetes. The mean values and prevalence of certain variables were calculated by diabetes status, standardised by 5-year age group, sex, and study area. Direct standardisation was used to calculate age-, sex-, and study area-adjusted disease incidence rates, using the total study population as the standard. Cox regression yielded hazard ratios (HR) for diabetes versus not, stratified by age-at-risk (5-year age groups), study area and, where appropriate, sex, and adjusted for education (no formal education, primary school, middle school, high school, college/university), smoking (never, occasional, ex-regular, current regular), alcohol (never, occasional intake, ex-regular, reduced intake, weekly intake), systolic blood pressure (SBP) (9 groups), and physical activity (5 groups). Sensitivity analyses were performed adjusting for age, SBP and physical activity as continuous variables.***  ***Comparison of HRs for the first four and subsequent years of follow-up revealed no evidence of departure from the proportional hazards assumption. Adjusted HRs were calculated across strata of other cardiovascular risk factors and duration of diabetes at baseline, and chi-square tests for trend and heterogeneity (ie, effect modification or statistical interaction) were applied to the log hazard ratios and their standard errors. In separate analyses, risk estimates were also adjusted for adiposity. Finally, the HR for MOVD associated with numbers of other presenting baseline cardiovascular risk factors (hypertension, overweight or obese, ever regular smoking, physical inactivity) among individuals with diabetes was assessed using the floating absolute risk method, which provides estimates of variance across all exposure categories.***  ***Population-attributable risk is P(HR-1)/(1+P[HR-1]), where P is prevalence of diabetes. By applying age-specific HRs (that can be assumed to approximate to the relative risk) to age-specific, nationally representative prevalences of diabetes and national cause-specific mortality data, we estimated the number of cardiovascular deaths attributable to diabetes.” Methods, paragraphs seven to ten.*** |
|  |  | (*b*) Describe any methods used to examine subgroups and interactions  ***Done.***  ***Methods used to examine subgroups and interactions are described in the “Statistical analyses” section of the Methods: “Adjusted HRs were calculated across strata of other cardiovascular risk factors and duration of diabetes at baseline, and chi-square tests for trend and heterogeneity (ie, effect modification or statistical interaction) were applied to the log hazard ratios and their standard errors”. Methods, paragraph nine.*** |
|  |  | (*c*) Explain how missing data were addressed  ***Done.***  ***Participants with missing data for key variables were excluded as described in the “Statistical analysis” section of the Methods (paragraph seven): “A further 1081 (0.2%) participants with missing, implausible or extreme values for blood pressure, height, waist circumference, hip circumference, waist-to-hip ratio or body mass index (BMI) were excluded”. Missing data are described for simple descriptive analyses in Table 1’s legend. There were no other missing data for the variables included in the main analyses.*** |
|  |  | (*d*) If applicable, explain how loss to follow-up was addressed  ***Done.***  ***Loss to follow-up is described in the “Follow-up for morbidity and mortality” section of the Methods: “By 1st January 2014, 25 488 (5.0%) participants had died and 2411 (0.5%) were lost to follow-up.” Methods, paragraph six.*** |
|  |  | (*e*) Describe any sensitivity analyses  ***Done.***  ***Sensitivity analyses are described in the “Statistical analyses” section of the Methods: “Sensitivity analyses were performed adjusting for age, SBP and physical activity as continuous variables” and “In separate analyses, risk estimates were also adjusted for adiposity”. Methods, paragraphs eight and nine.*** |
| Results | | |
| Participants | 13* | (a) Report numbers of individuals at each stage of study—eg numbers potentially eligible, examined for eligibility, confirmed eligible, included in the study, completing follow-up, and analysed  ***Done.***  ***The participation rate and number of individuals recruited to the study are discussed in the “Study population” section of the Methods (paragraph two): “Overall, ~30% responded, and 512 891 individuals were enrolled”. Loss to follow-up is described in the “Follow-up for mortality and morbidity” section of the Methods (paragraph six): “…2411 (0.5%) were lost to follow-up”. Participant exclusions are described in the “Statistical analysis” section of the Methods (paragraph seven): “The main analyses excluded participants reporting prior doctor-diagnosed IHD (n=15 472, 3.0%) or stroke/transient ischaemic attack (n=8884, 1.7%) at baseline. A further 1081 (0.2%) participants with missing, implausible or extreme values for blood pressure, height, waist circumference, hip circumference, waist-to-hip ratio or body mass index (BMI) were excluded, leaving 488 760 (199 896 men, 288 864 women) for the present analyses”.*** |
|  |  | (b) Give reasons for non-participation at each stage  ***Done.***  ***Reasons for non-participation at each stage are described in the “Follow-up for mortality and morbidity” section of the Methods (paragraph six): “…2411 (0.5%) were lost to follow-up”; and in the “Statistical analysis” section of the Methods (paragraph seven): “The main analyses excluded participants reporting prior doctor-diagnosed IHD (n=15 472, 3.0%) or stroke/transient ischaemic attack (n=8884, 1.7%) at baseline. A further 1081 (0.2%) participants with missing, implausible or extreme values for blood pressure, height, waist circumference, hip circumference, waist-to-hip ratio or body mass index (BMI) were excluded, leaving 488 760 (199 896 men, 288 864 women) for the present analyses”.*** |
|  |  | (c) Consider use of a flow diagram  ***A flow diagram has not been used but figures are presented in the text, as described in Sections 13(a) and 13(b).*** |
| Descriptive data | 14* | (a) Give characteristics of study participants (eg demographic, clinical, social) and information on exposures and potential confounders  ***Done.***  ***Characteristics of study participants are presented in Table 1 and in the text of the Results section: “Among 488 760 participants without prior cardiovascular diseases, the mean (SD) baseline age was 51 (11) years, 2.7% (n=13 284) reported a history of doctor-diagnosed diabetes and a further 2.7% (n=13 051) had screen-detected diabetes, of whom 8554 (65.5%) were identified through RPG measurement (RPG ≥7.0 mmol/L and time since last food ≥8 hours: n=3986; RPG ≥11.1 mmol/L and time since last food <8 hours: n=4568), 1447 (11.1%) through fasting plasma glucose measurement and 3050 (23.4%) through both. For both self-reported and screen-detected diabetes the prevalence was slightly higher in women than in men (2.9% versus 2.5% and 2.8% versus 2.6%, respectively), mainly at ages >50 years (S2 Fig) and in urban than in rural areas (3.9% versus 1.8% and 3.4% versus 2.1%, respectively). Participants reporting a history of diabetes were older, less likely to be current smokers or alcohol drinkers, but more likely to be ex-smokers or ex-drinkers and to be physically inactive than those without diabetes, and had an approximately four-fold greater prevalence of a family history of diabetes (Table 1). Those with screen-detected diabetes were older and about twice as likely to have a family history of diabetes as those without diabetes, but had comparable smoking and alcohol consumption patterns. The prevalence of obesity and hypertension were higher in participants with diabetes, particularly screen-detected diabetes. Among participants with self-reported diabetes, the median age at diagnosis was 53 (range 4 to 77) years, and the median duration of diabetes at baseline was 4 (0 to 56) years, with 76% (n=8501) reporting use of insulin or oral hypoglycaemic agents.” Results, paragraph one.*** |
|  |  | (b) Indicate number of participants with missing data for each variable of interest  ***Done.***  ***The number of participants with missing data for each variable is presented in the Table 1 legend.*** |
|  |  | (c) Summarise follow-up time (eg, average and total amount)  ***Done.***  ***Follow-up time is summarised in the second paragraph of the Results section: “During ~3.4 million person-years (mean 7 years) of follow-up…”. Results, paragraph two.*** |
| Outcome data | 15* | Report numbers of outcome events or summary measures over time  ***Done.***  ***The numbers of cardiovascular disease outcomes are presented in Table 2.*** |
| Main results | 16 | (*a*) Give unadjusted estimates and, if applicable, confounder-adjusted estimates and their precision (eg, 95% confidence interval). Make clear which confounders were adjusted for and why they were included  ***Estimates adjusted for age, sex and study area only are presented in Table 2 and S3 Table.*** |
|  |  | (*b*) Report category boundaries when continuous variables were categorized  ***Done.***  ***The main exposure is dichotomous (diabetes) but blood glucose categories used to define screen-detected diabetes are presented in the “Assessment of diabetes status” section of the Methods: “Screen-detected diabetes was defined as not having self-reported diabetes, but having a random plasma glucose level ≥7.0mmol/L if time since last food ≥8 hours, or ≥11.1mmol/L if time since last food <8 hours, or a fasting plasma glucose level ≥7.0mmol/L on subsequent testing.” Methods, paragraph four.*** |
|  |  | (*c*) If relevant, consider translating estimates of relative risk into absolute risk for a meaningful time period  ***Done.***  ***Cardiovascular disease rates in individuals with and without diabetes are presented in Table 2, S1 Table and S3 Table.*** |
| Other analyses | 17 | Report other analyses done—eg analyses of subgroups and interactions, and sensitivity analyses  ***Done. Findings of multiple subgroup analyses are presented throughout the Results section: “For IS and MOVD (S4 Fig), the associations were stronger at younger ages (both p for trend <0.001) and in rural areas (p for heterogeneity = 0.003 and <0.001, respectively). The risk of MCE varied little across population subgroups” (Results, paragraph two); “Self-reported diabetes was also associated with significant, but more modest, excess risk of ICH…… The risk varied little across participant subgroups (S5 Fig)” (Results, paragraph three); “Among participants with screen-detected diabetes, the excess risks were also highly significant….. There was little evidence of heterogeneity in the observed HRs for MCE, ICH (S6 Fig) or cardiovascular mortality (S7 Fig) across population subgroups. For IS and MOVD (S8 Fig), however, the risk was somewhat greater at younger ages (both p for trend <0.001), in rural residents (p for heterogeneity <0.001 and 0.003, respectively), and in more physically active individuals (p for trend 0.008 and 0.006, respectively)” (Results, paragraph five). Results of sensitivity analyses are also reported: “Additional adjustment for waist-to-hip ratio modestly, but non-significantly, attenuated HRs for ischaemic cardiovascular diseases, but not for ICH or cardiovascular mortality (S1 Table). In sensitivity analyses excluding participants who developed incident diabetes during follow-up (n= 8896) these HRs remained largely unchanged (S2 Table). Sensitivity analyses adjusting for age, SBP and physical activity as continuous variables also produced the same findings.” (Results, paragraph four)*** |
| Discussion | | |
| Key results | 18 | Summarise key results with reference to study objectives  ***Done.***  ***The key results are summarised in the first paragraph of the Discussion: “It showed that self-reported, doctor-diagnosed diabetes was associated with 1.5- to 2.5-fold increased risks of cardiovascular mortality and incident IHD and IS. Moreover, it provided strong prospective evidence of a significant, though more modest, adverse effect of diabetes on ICH risk. Individuals with screen-detected diabetes also had significantly increased risks.”*** |
| Limitations | 19 | Discuss limitations of the study, taking into account sources of potential bias or imprecision. Discuss both direction and magnitude of any potential bias  ***Done.***  ***Limitations of the study are described throughout the Discussion where relevant, including: “The method of ascertainment of IHD in the CKB may misclassify a proportion of individuals with “silent” myocardial ischaemia, more prevalent among individuals with diabetes, leading to underestimation of diabetes-associated IHD risk” (Discussion, paragraph two); “The more modest effects of screen-detected, than self-reported, diabetes may reflect shorter duration of, or less severe glycaemic aberration in, screen-detected diabetes, and greater potential for misclassification” (Discussion, paragraph two); “Although self-reported diabetes is widely used, it may be subject to misclassification” (Discussion, paragraph six); “We did not collect specific information on diabetes type” (Discussion, paragraph six); “Separate examination of screen-detected diabetes…may be subject to greater misclassification resulting from use of RPG measurement on a glucometer for diagnosis…and would result in underestimates of diabetes-associated risks” (Discussion, paragraph seven); “Data on lipids are not currently available; this may have resulted in residual confounding, although progressive adjustment for confounders in one published study would suggest this would be minimal. Similarly, renal function data are not currently available in the CKB; this precludes investigation of effect modification of the association of diabetes with cardiovascular diseases but would not bias our risk estimates” (Discussion, paragraph seven).*** |
| Interpretation | 20 | Give a cautious overall interpretation of results considering objectives, limitations, multiplicity of analyses, results from similar studies, and other relevant evidence  ***Done.***  ***Overall interpretation of the results considering objectives, limitations, multiplicity of analyses, results from similar studies, and other relevant evidence is provided throughout the Discussion, for example, “Our study provides the first large-scale prospective evidence of the cardiovascular consequences of diabetes among adults in China. It showed that self-reported, doctor-diagnosed diabetes was associated with 1.5- to 2.5-fold increased risks of cardiovascular mortality and incident IHD and IS. Moreover, it provided strong prospective evidence of a significant, though more modest, adverse effect of diabetes on ICH risk. Individuals with screen-detected diabetes also had significantly increased risks. If these associations were causal, then almost 0.5 million cardiovascular deaths a year in China could now be attributed to diabetes”. Discussion, paragraph one.*** |
| Generalisability | 21 | Discuss the generalisability (external validity) of the study results  ***Done.***  ***The generalisability of the study results to the Chinese population as a whole and to non-Chinese populations is discussed at multiple points in the Discussion section: “Previous studies of predominantly Western populations have shown approximate doubling of IHD risk in diabetes. The comparability of our risk estimates with previous studies suggests differences between East Asian and Western populations in the relative importance of insulin resistance and beta-cell dysfunction in the aetiology of diabetes have little, if any, impact on associated IHD risk” (Discussion, paragraph two); “The association of screen-detected diabetes with IS in our study is largely consistent with data from studies of mostly Western populations, while the association with self-reported diabetes was moderately weaker than in those studies” (Discussion, paragraph three); “Although not designed to be nationally representative, given the size and diversity of the CKB study population and minimal loss to follow-up, this would not be expected to bias risk estimates or reduce their generalisability to the Chinese adult population. Furthermore, the prevalence of diabetes in the CKB was similar to that reported in a reasonably contemporaneous, representative Chinese survey in 2000-01. More recent surveys have reported a higher rate of diabetes in China, with, for example, a prevalence rate of 11.6% in the 2010 survey; as well as secular trends, the lower prevalence of diabetes in the CKB may reflect different study settings, sampling methods and approaches used to identify undiagnosed diabetes. The prevalence of self-reported diabetes was, however, similar (3.5% versus 2.7% in the present CKB population and 3.2% in the CKB population including individuals with prior cardiovascular disease)” (Discussion, paragraph six); “The present nationwide prospective study provides the first large-scale evidence from mainland China that individuals with diabetes are at significantly increased risk of major cardiovascular diseases, similar in magnitude to that observed in Western populations” (Discussion, paragraph eight).*** |
| Other information | | |
| Funding | 22 | Give the source of funding and the role of the funders for the present study and, if applicable, for the original study on which the present article is based  ***Done.***  ***The sources of funding are described in the “Funding” section: “The baseline survey and the first re-survey were supported by a research grant from the Kadoorie Charitable Foundation in Hong Kong. The long-term continuation of the project is supported by program grants from the UK Wellcome Trust (088158/Z/09/Z, 104085/Z/14/Z); the Chinese Ministry of Science and Technology (2011BAI09B01, 2012-14); and the Chinese National Natural Science Foundation (81390541). The British Heart Foundation; UK Medical Research Council; and Cancer Research UK provide core funding to the Oxford CTSU. Fiona Bragg acknowledges support from the BHF Centre of Research Excellence, Oxford.”*** |

*Give information separately for exposed and unexposed groups.
